# Repositioning Antimicrobial Peptides Against WHO‐Priority Fungi

**DOI:** 10.1002/advs.202509567

**Published:** 2025-08-30

**Authors:** Cesar Augusto Roque‐Borda, Kaila Petronila Medina‐Alarcón, João Paulo Soler Gonçalves Pereira, Thais Cristina dos Anjos Sevilhano, Brigitte Aguilar‐Morón, Fernando Díaz‐Cárdenas, Lucas Silva da Cruz, Francisco Humberto Xavier‐Júnior, Eduardo Festozo Vicente, João Perdigão, Beatriz G. de la Torre, Fernando Albericio, Fernando Rogério Pavan

**Affiliations:** ^1^ Department of Biological Sciences School of Pharmaceutical Sciences Universidade Estadual Paulista (UNESP) Araraquara 14800‐903 Brazil; ^2^ iMed.ULisboa–Institute for Medicines Research Faculty of Pharmacy University of Lisbon Lisbon 1649004 Portugal; ^3^ Department of Clinical Analysis School of Pharmaceutical Sciences Universidade Estadual Paulista (UNESP) Araraquara 14800‐903 Brazil; ^4^ School of Sciences and Engineering São Paulo State University (UNESP) Tupã 17602‐496 Brazil; ^5^ Vicerrectorado de Investigación Universidad Católica de Santa María Arequipa 04000 Peru; ^6^ Laboratory of Pharmaceutical Biotechnology (BioTecFarm) Department of Pharmaceutical Sciences Federal University of Paraiba Campus Universitário I, Castelo Branco III. Cidade Universitária. CEP João Pessoa‐PB 58051–900 Brazil; ^7^ School of Laboratory Medicine and Medical Sciences College of Health Sciences University of KwaZulu‐Natal Durban 4041 South Africa; ^8^ School of Chemistry and Physics University of KwaZulu‐Natal Durban 4041 South Africa; ^9^ Department of Organic Chemistry University of Barcelona Barcelona 08028 Spain

**Keywords:** antimicrobial peptides, fungal infections, multidrug‐resistant fungi, target delivery

## Abstract

The growing threat of fungal infections, particularly in immunocompromised individuals, is exacerbated by the limited number of antifungal drug classes, increasing resistance rates, and complex hostpathogen interactions. In response to this public health concern, the World Health Organization published its first list of fungal priority pathogens, including C. auris, A. fumigatus, C. neoformans, and C. albicans. These species exhibit multidrug resistance, virulence plasticity, and enhanced biofilm‐forming capacity, which contributes to antifungal tolerance and complicates treatment outcomes. Antimicrobial peptides (AMPs) have emerged as promising alternatives due to their broad‐spectrum activity, rapid membrane‐disrupting mechanisms, and low propensity to induce resistance. This review provides an in‐depth analysis of AMP‐based antifungal strategies, integrating insights from structureactivity relationships, molecular engineering, and targeted delivery systems. Strategies such as peptide hybridization, cyclization, PEGylation, and nanoparticle conjugation are examined to enhance stability, specificity, and pharmacokinetics. Opportunities for rational AMP design are also discussed, leveraging computational toolsincluding machine learning and deep learning approachesalongside immunoproteomic targeting. Together, these multidisciplinary advances underscore the potential of AMPs as next‐generation therapeutics against critical fungal pathogens. Nonetheless, clinical translation remains challenging, requiring continued investment in formulation science, regulatory alignment, and translational development pipelines.

## Graphical abstract

The growing burden of drug‐resistant fungal infections, driven by pathogens such as *Candida auris, Cryptococcus neoformans*, and *Aspergillus fumigatus*, underscores the urgent need for novel antifungal therapies. This review explores antimicrobial peptides as promising agents with membrane‐disruptive activity, immunomodulatory properties, and delivery potential, focusing on their role against the World Health Organization's priority fungal pathogens.

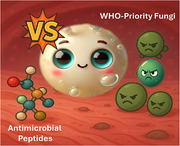

## 1 Introduction

Fungal infections have become an increasingly serious global health concern, particularly in the context of immunocompromised populations, intensive care settings, and the growing use of immunosuppressive therapies.^[^ ^1^, ^2^ ^]^ In 2022, the World Health Organization (WHO) released the first‐ever fungal priority pathogens list, underscoring the emergence of species such as *Candida auris, Cryptococcus neoformans, Aspergillus fumigatus*, and *Candida albicans* as critical threats due to their high mortality rates, multidrug resistance (MDR), and persistent nosocomial transmission.^[^ ^3^ ^]^ Despite the availability of major antifungal classes—azoles, echinocandins, polyenes, allylamines, and antimetabolites—the therapeutic landscape remains alarmingly limited.^[^ ^4^ ^]^ These agents often exhibit toxicity, poor bioavailability, and predominantly fungistatic activity, which may be insufficient for pathogen clearance in immunocompromised hosts.^[^ ^5^ ^]^ Moreover, biofilm formation, phenotypic plasticity, and host immune evasion further complicate effective management, particularly in invasive candidiasis, aspergillosis, and cryptococcosis.^[^ ^6^, ^7^, ^8^ ^]^

In light of these limitations, AMPs have gained substantial interest as a novel therapeutic modality.^[^ ^9^, ^10^ ^]^ These evolutionarily conserved molecules, found in virtually all life forms, possess potent antifungal activity, often through mechanisms that bypass traditional drug targets, such as direct membrane disruption, mitochondrial dysfunction, ion imbalance, and immune modulation.^[^ ^11^ ^]^ Recent developments in AMP design, including rational sequence engineering, peptidomimetic strategies, conjugation with antifungal drugs or nanocarriers, and targeted delivery platforms, have significantly enhanced their pharmacological profiles and clinical potential.^[^ ^12^, ^13^ ^]^

This review provides a comprehensive and integrative analysis of AMP‐based antifungal strategies, aligned with the WHO fungal priority pathogen list. Virulence and resistance mechanisms of critical fungal species, elucidate structure–activity relationships (SAR) of antifungal peptides, and assess emerging delivery technologies—including PEGylation, nanoparticle conjugation, and hybridization are examinated. Furthermore, we explore current preclinical and clinical efforts, and highlight opportunities for rational AMP design through computational tools—including machine learning (ML) and its subset, deep learning (DL). While ML encompasses a broad range of statistical and algorithmic approaches, DL refers specifically to neural network‐based models capable of extracting hierarchical patterns from complex biological data, such as AMP–fungi interaction matrices or sequence–activity relationships.

## 2 Virulence Factors of Critical Priority Fungi

**Figure 1:**
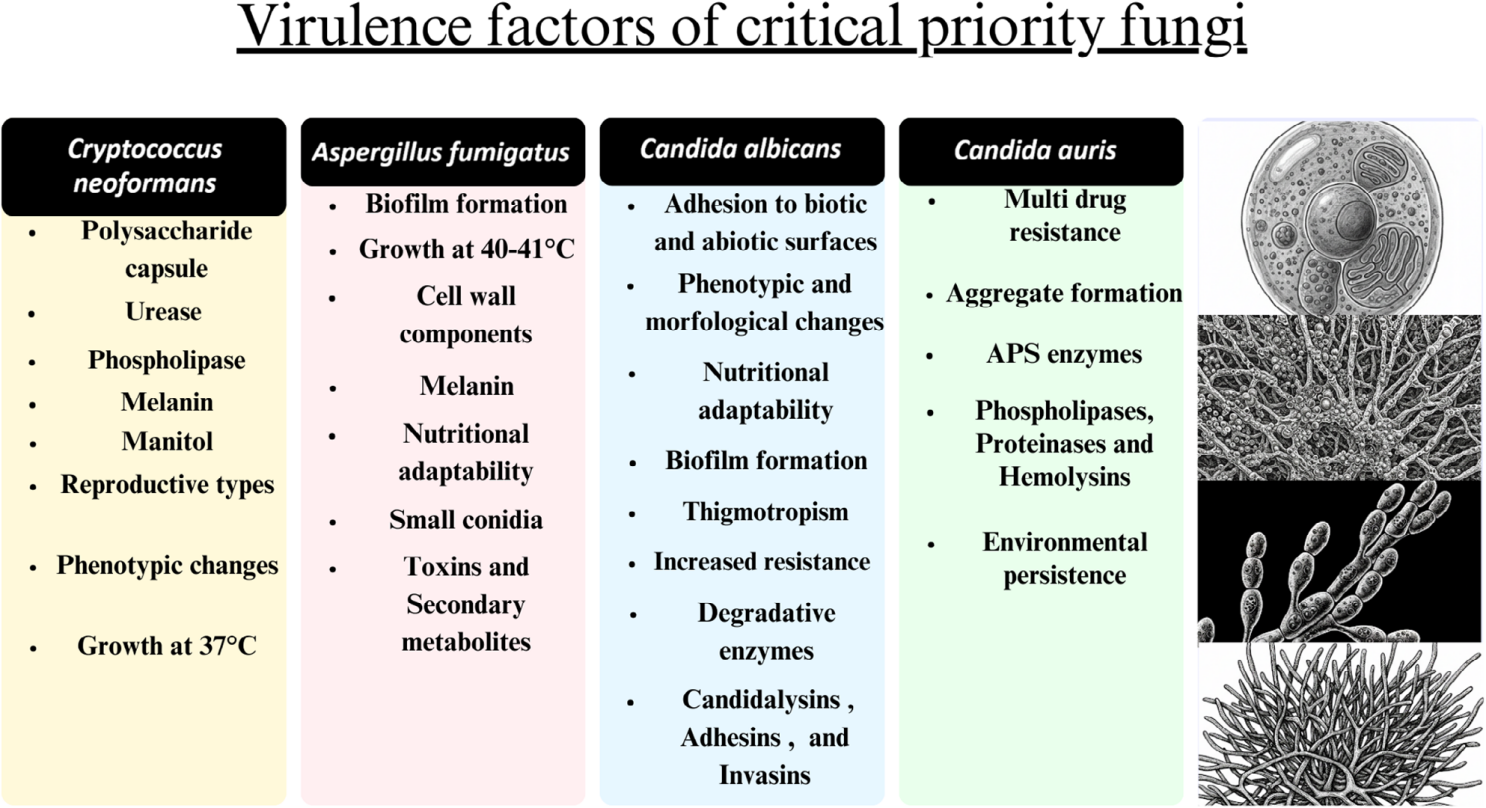
Virulence factors of WHO‐designated critical priority fungal pathogens. This figure summarizes the major virulence traits of C*ryptococcus neoformans, Aspergillus fumigatus, Candida albicans*, and *Candida auris*, including thermotolerance, biofilm formation, immune evasion mechanisms, enzyme production, and resistance features. The infographic was designed using Canva. Illustrative fungal diagrams were obtained via Gemini AI and are included for visual representation purposes only.

In general, virulence factors can be defined as deleterious tools of pathogenicity, whose function is to ensure the survival and adaptation of fungal organisms in hostile environments.^[^ ^14^, ^15^ ^]^ According to the WHO list of priority fungal pathogens released in 2022, *C. neoformans*, *A. fumigatus*, *C. auris*, and *C. albicans* have been classified as critical priority pathogens, primarily due to their remarkable adaptability, ability to evade host defenses, and growing resistance to antifungal agents.^[^ ^16^ ^]^ These fungi exhibit a wide array of virulence mechanisms, including biofilm formation, morphological transitions, secretion of hydrolytic enzymes, thermotolerance, and immune modulation, which contribute to their persistence and pathogenicity (**Figure** **1**). A detailed overview of the main virulence mechanisms shared by WHO‐designated critical‐priority fungi is presented in the following section. These include biofilm formation, morphological plasticity, thermotolerance, secretion of hydrolytic enzymes, and immune evasion—factors that collectively drive pathogenesis, persistence, and antifungal resistance, particularly in immunocompromised and critically ill patients.

### 2.1 C. neoformans

*C. neoformans* is a life‐threatening opportunistic pathogen with a multifactorial virulence profile that enables immune evasion, pulmonary colonization, and dissemination to the central nervous system (CNS) via blood–brain barrier penetration. Key virulence factors include a polysaccharide capsule, degradative enzymes (e.g., urease and phospholipase), melanin, mannitol, reproductive type variation, phenotypic switching, and thermotolerance at 37 °C.^[^ ^17^, ^18^ ^]^ The polysaccharide capsule is the major virulence determinant and is composed mainly of glucuronoxylomannanagalactan (GXMGal), galactoxylomannan (GalXM), and mannoproteins.^[^ ^19^, ^20^, ^21^ ^]^ GXMGal and GalXM possess immunomodulatory activities and can impair macrophage function. The capsule also inhibits phagocytosis in the absence of opsonins and protects fungal cells against oxidative stress.^[^ ^21^, ^22^, ^23^ ^]^

Degradative enzymes such as urease and phospholipase facilitate CNS invasion. Urease hydrolyzes urea into carbon dioxide and ammonia, not only supplying nitrogen but also contributing to microvascular epithelial damage and enhancing fungal penetration into the blood‐brain barrier.^[^ ^24^, ^25^, ^26^, ^27^ ^]^ Phospholipase disrupts cell membranes and tight junctions, promoting translocation across endothelial barriers.^[^ ^19^, ^23^ ^]^ Melanin is another important virulence factor. This brown/black hydrophobic pigment is synthesized using host catecholamines and enhances resistance to oxidants, acids, and alkalis. Melanin protects the fungus during phagocytosis and is implicated in the neurotropism of *C. neoformans*.^[^ ^28^, ^29^, ^30^, ^31^ ^]^ Mannitol plays a similar protective role by scavenging reactive oxygen species (ROS).^[^ ^23^, ^32^ ^]^

Over time, antifungal resistance in *C. neoformans* has become a growing concern. Resistance to azoles, especially fluconazole, is increasingly reported, although amphotericin B remains the first‐line treatment for systemic cryptococcosis.^[^ ^33^ ^]^ Fluconazole resistance involves multiple mechanisms, such as ERG11 mutations, altered stress response pathways, efflux pump upregulation, and membrane trafficking alterations.^[^ ^34^ ^]^ In particular, the *cnAFR1* gene encodes an ABC transporter that actively exports fluconazole, lowering its intracellular concentration and promoting resistance.^[^ ^35^ ^]^ This efflux pump also enhances macrophage resistance, favoring persistent infections.^[^ ^36^ ^]^

Notably, capsule plasticity contributes to antifungal resistance. The capsule can enlarge and increase the production of GXM, GalXM, and mannoproteins, reinforcing protection against oxidative stress, radiation, desiccation, and phagocytosis.^[^ ^21^, ^37^, ^38^, ^39^, ^40^ ^]^ An emerging resistance mechanism involves polyploid Titan cells, which are enlarged cryptococcal cells generated during infection. These cells overexpress genes linked to stress adaptation and capsule/cell wall synthesis, including transcription factors such as *Stb4, Zfc3*, and *Bzp4*.^[^ ^41^, ^42^ ^]^ Titan cell progeny exhibit enhanced fluconazole resistance, attributed to structural adaptations that protect against ROS and phagocytic killing.^[^ ^43^, ^44^ ^]^

### 2.2 A. fumigatus

*A. fumigatus* is a thermotolerant, filamentous fungus capable of thriving at 40–41 °C and its virulence is driven by multiple factors, including nutritional adaptability, immune evasion, and a strong ability to form biofilms, which enable persistence in polymicrobial niches.^[^ ^45^ ^]^ Among its most potent virulence traits is the production of melanized conidia (2–3µm in diameter), as well as a diverse array of secondary metabolites and toxins. These compounds—such as gliotoxins, aflatoxins, fumagillins, helvolic acid, and ribotoxins (e.g., restrictocin and mitogillin)—are key to the pathogen's immune modulation, phagocytosis avoidance, and host tissue colonization.^[^ ^46^, ^47^, ^48^, ^49^, ^50^, ^51^, ^52^ ^]^ The cell wall of *A. fumigatus* is enriched with structural polysaccharides including β‐1,3‐glucan, β‐1,4‐glucan, α‐1,3‐glucan, chitin, mannans, galactomannans, and chitosan, which provide rigidity while concealing pathogen‐associated molecular patterns. This structural barrier not only impairs host immune detection but also facilitates biofilm establishment, a process central to antifungal resistance.^[^ ^53^, ^54^, ^55^, ^56^, ^57^, ^58^, ^59^ ^]^

Biofilm‐associated *A. fumigatus* exhibits elevated minimal inhibitory concentrations (MICs) compared to planktonic cells, a phenomenon attributed to extracellular matrix components (e.g., galactosaminogalactan), efflux pumps (AfuMDR1–4), eDNA, persister cells, and molecular chaperones like Hsp90.^[^ ^47^, ^60^, ^61^ ^]^ The Hsp90–calcineurin and HOG–MAPK signaling pathways further support resistance and adaptation under environmental stress. Recent findings have revealed that *A. fumigatus* produces oxylipins—oxidized fatty acid derivatives—that modulate both fungal development and host–pathogen interactions. In particular, the oxylipin 5,8‐diHODE, synthesized via the fungal oxygenase PpoA and regulated by the transcription factor ZfpA, was shown to protect *A. fumigatus* against hyphal tip damage induced by echinocandins. This signal also promotes hyphal branching and differentiation, contributing to tissue colonization and immune evasion. Interestingly, 5,8‐diHODE and related oxylipins share structural similarities with mammalian lipid mediators, and may interact with host G‐protein coupled receptors such as G2A. Indeed, G2A‐deficient mice exhibit enhanced neutrophil recruitment and better survival during invasive aspergillosis, suggesting that these fungal molecules may dampen immune responses to favor fungal persistence.^[^ ^62^ ^]^

**Figure 2:**
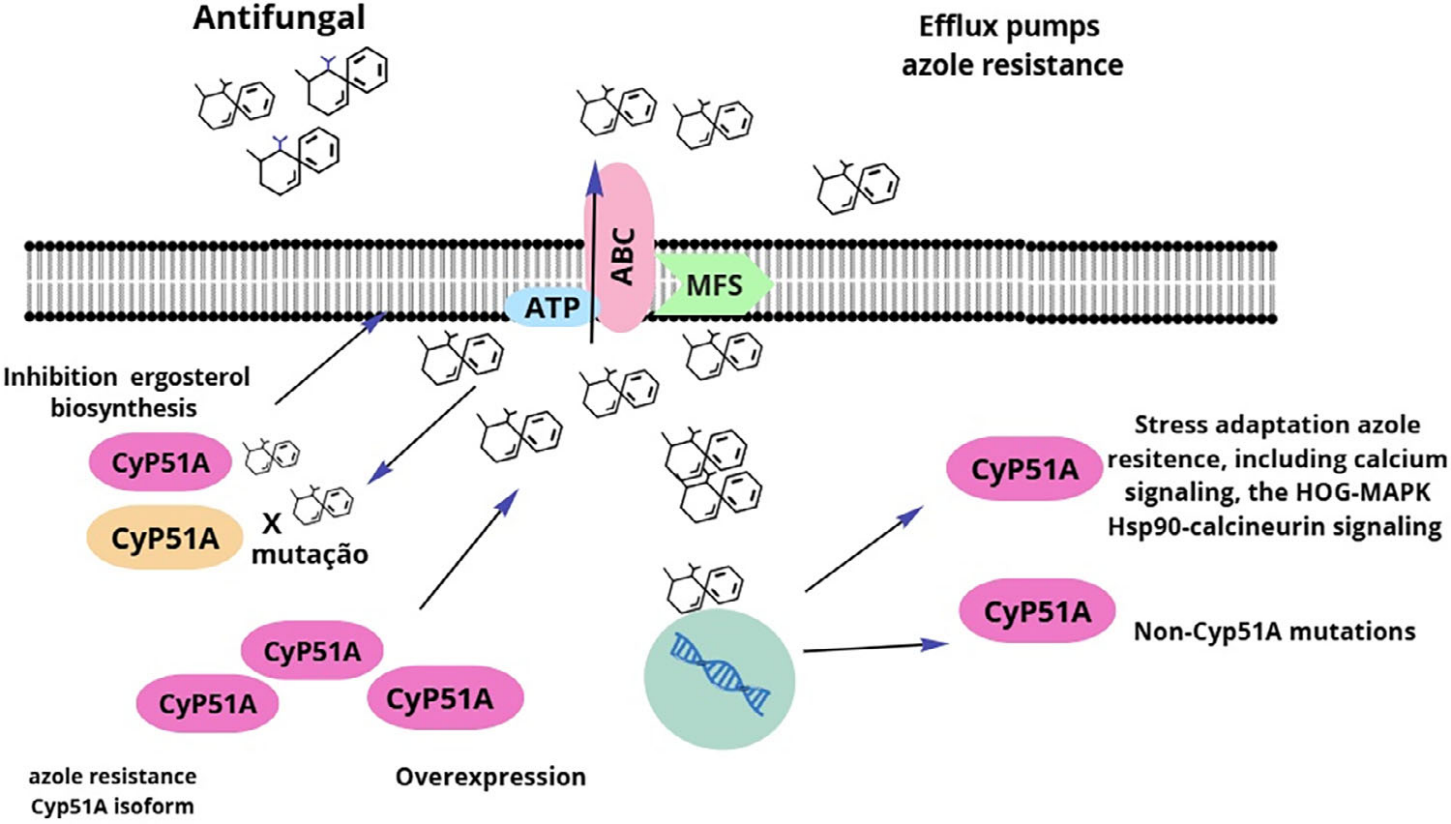
Molecular mechanisms of azole resistance in *Aspergillus fumigatus*. Azole resistance involves multiple mechanisms, including mutations and overexpression of the Cyp51A gene, which encodes the target enzyme of azoles involved in ergosterol biosynthesis. Additional mechanisms include the action of efflux pumps—particularly ATP‐binding cassette (ABC) and major facilitator superfamily (MFS) transporters—that actively expel azole compounds from the cell. Resistance is further supported by non‐Cyp51A mutations and stress‐response pathways such as calcium signaling, HOG‐MAPK, and Hsp90–calcineurin signaling cascades.

One of the most critical clinical challenges is the emergence of azole resistance, with global prevalence rates ranging from 6.6% to 28%, depending on geographical region.^[^ ^63^, ^64^ ^]^ Resistance is frequently associated with biofilm formation, which restricts drug penetration and reduces therapeutic efficacy.^[^ ^47^, ^65^, ^66^ ^]^ At the molecular level, azole resistance is predominantly mediated by mutations in the *Cyp51A* gene, which encodes a 14α‐demethylase enzyme crucial for ergosterol biosynthesis.^[^ ^67^, ^68^, ^69^ ^]^ Two isoforms, Cyp51A and Cyp51B, have been described, though resistance is largely attributed to Cyp51A. In particular, tandem repeat (TR) mutations in the Cyp51A promoter region—such as TR34/L98H, TR46/Y121F/T289A, and TR53—lead to gene overexpression and increased resistance. These variants are prevalent in both clinical and environmental settings, indicating widespread strain dissemination.^[^ ^70^, ^71^ ^]^ Additional mechanisms include: Overexpression of efflux transporters, such as ABC (e.g., AfuMDR1–4) and MFS‐type transporters, which reduce intracellular azole concentrations;^[^ ^72^, ^73^, ^74^ ^]^ non‐Cyp51A mutations, such as deletions in *afyap1* and *aldA*, and point mutations like R243Q in AfCox10, affecting sterol synthesis;^[^ ^75^, ^76^ ^]^ and activation of stress response pathways, including calcium signaling, iron regulation, cell wall integrity, and the Hsp90–calcineurin axis (**Figure** **2**).^[^ ^77^, ^78^, ^79^, ^80^, ^81^ ^]^

Biofilm‐related resistance in *A. fumigatus* is multifactorial, involving ECM overproduction, cell dormancy, upregulation of efflux genes like *mdr4, cdr1B*, and *mdr1*,^[^ ^82^, ^83^, ^84^, ^85^, ^86^ ^]^ and retention of eDNA and GAG, which sustain biofilm architecture. Enzymatic degradation of GAG by Sph3 has been shown to potentiate the activity of caspofungin and other antifungals.^[^ ^87^ ^]^ Given the complexity of these resistance mechanisms, novel therapeutic strategies are urgently needed. Promising candidates include antifungal peptides such as hLF(1–11) and dhvar5, which can act against conidia without causing cytotoxicity to red blood cells.^[^ ^88^ ^]^ Additionally, synergistic approaches, such as combining caspofungin with polymyxin B, have shown efficacy against mixed‐species biofilms involving *A. fumigatus* and *Pseudomonas aeruginosa*, suggesting that combination therapies may offer renewed clinical value.^[^ ^83^ ^]^

### 2.3 C. auris

*C. auris* has emerged as a multidrug‐resistant fungal pathogen, distinguished by a unique set of virulence attributes. These include aggregate formation, secretion of hydrolytic enzymes, remarkable environmental persistence, and resistance to all three major antifungal classes: azoles, polyenes, and echinocandins.^[^ ^89^, ^90^ ^]^ A notable feature of this pathogen is its tendency to form aggregates of undivided daughter cells, resulting in clusters that exhibit enhanced resistance to antifungal agents. Unlike *C. albicans, C. auris* does not typically form true hyphae or pseudohyphae, a difference likely attributed to the absence of Candidalysin, a hyphal‐specific virulence factor.^[^ ^91^, ^92^, ^93^ ^]^ Although it produces less biofilm than *C. albicans*, the quantity is still sufficient to support pathogenicity.^[^ ^94^ ^]^ Furthermore, host immune responses appear compromised, as neutrophil recruitment during *C. auris* infection is significantly reduced—by ≈50%—which may impair fungal clearance.^[^ ^95^, ^96^ ^]^

This pathogen secretes a range of degradative enzymes, such as secreted aspartyl proteinases (SAPs), phospholipases, and hemolysins, which play a role in host tissue invasion, immune evasion, and adhesion.^[^ ^97^, ^98^ ^]^ However, the expression of these virulence factors can vary among isolates. One of the most alarming traits of *C. auris* is its robust environmental survival, persisting for extended periods on dry or damp surfaces, facilitating nosocomial transmission and underscoring the need for strict infection control measures.^[^ ^99^, ^100^, ^101^ ^]^ *C. auris* causes severe invasive infections, including candidemia and sepsis, with reported mortality rates reaching up to 72%. Resistance is mediated by multiple mechanisms, including mutations in drug targets, transcriptional upregulation of resistance genes, and increased efflux activity, all of which reduce intracellular antifungal concentrations.^[^ ^95^, ^96^, ^102^, ^103^ ^]^

Alarmingly, ≈90% of *C. auris* isolates are resistant to at least one antifungal class, and between 30% and 41% exhibit resistance to all three main antifungal categories, reflecting its highly MDR phenotype.^[^ ^104^, ^105^, ^106^ ^]^ While the mechanisms behind amphotericin B resistance remain unclear, they have been linked to alterations in ergosterol biosynthesis, including upregulation of ERG1, ERG2, ERG6, and ERG13.^[^ ^107^ ^]^ Azole resistance correlates with ERG11 gene amplification, single nucleotide polymorphisms, and overexpression of ABC and MFS‐type efflux pumps.^[^ ^108^, ^109^, ^110^ ^]^ Echinocandin resistance in *C. auris* has been attributed to mutations in the FKS1 gene, particularly at S639 within Hotspot 1, impairing the function of β‐1,3‐glucan synthase and thereby reducing drug‐binding efficiency.^[^ ^111^ ^]^

To further elucidate the interaction between *C. auris* and host barriers, ex vivo skin models developed by Seiser et al.^[^ ^111^ ^]^ demonstrated that *C. auris* fails to penetrate intact human epidermis but readily colonizes damaged skin—especially via hair follicles or mechanically disrupted sites. During dermal invasion, a shift toward pseudohyphal morphology was observed, suggesting an adaptive response to the microenvironment and emphasizing the importance of skin integrity in preventing fungal spread. In vivo studies by Areitio et al.^[^ ^112^ ^]^ support the heightened virulence of non‐aggregative *C. auris* strains, which exhibited increased renal pathology, granulomatous inflammation, and a greater fungal burden in the kidneys and spleen, when compared to aggregative variants.

**Figure 3:**
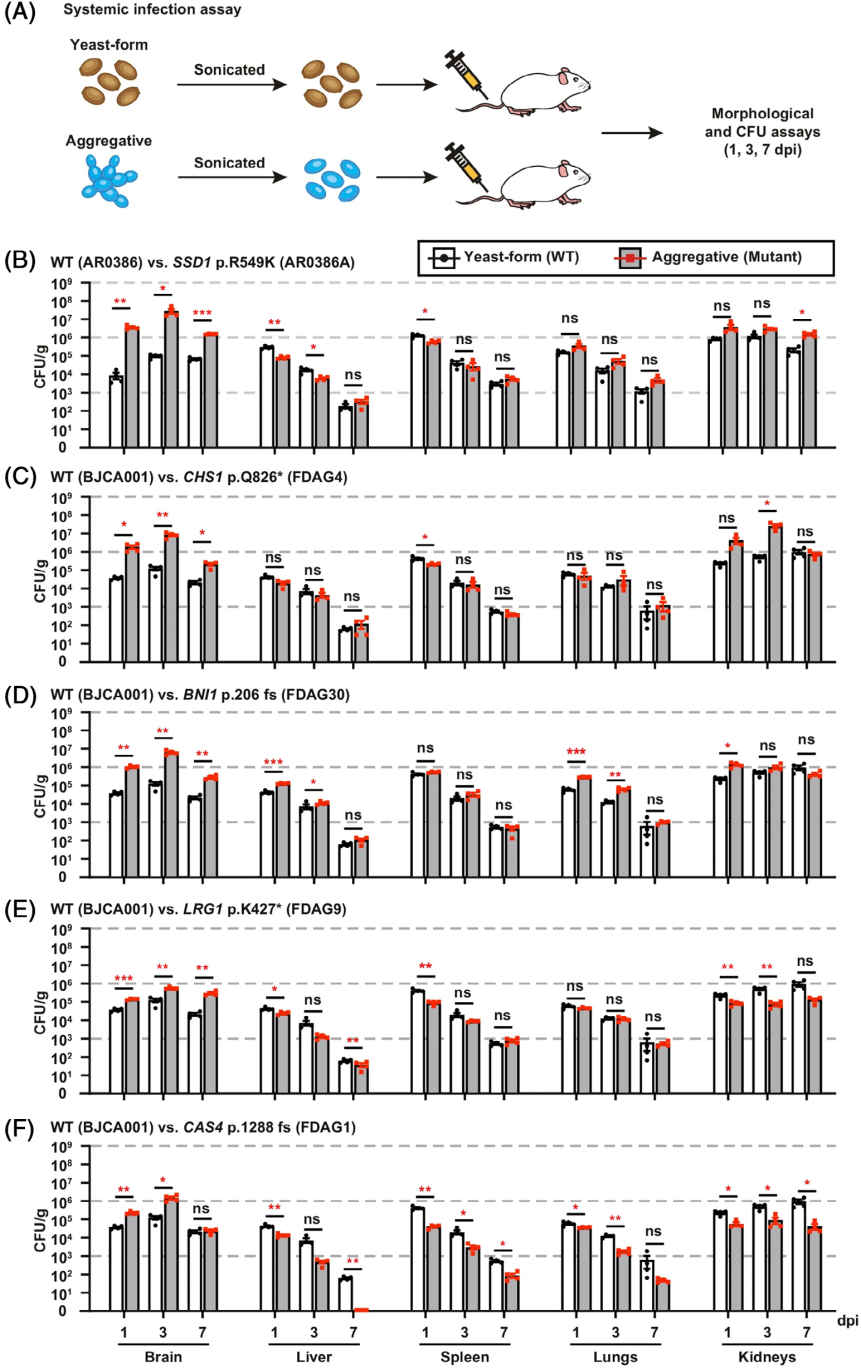
Systemic infection model and organ fungal burden in mice infected with WT or mutant *C. auris*. A) Yeast‐form and aggregative cells were sonicated and injected (1 × 10⁷ cells per mouse). Mice were euthanized at 1, 3, or 7 dpi; CFUs were measured in major organs. B–F) Fungal burden (CFU/g) in brain, liver, spleen, lungs, and kidneys from mice infected with: B) AR0386 vs. SSD1 mutant; C–F) BJCA001 vs. CHS1, BNI1, LRG1, or CAS4 mutants. Bars: mean ± SD (n = 4). Stats: paired two‐tailed t‐test (*P* < 0.05, *P *< 0.01, *P* < 0.001, ns = not significant). Reproduced with permission.^[^ ^113^ ^]^ Copyright 2024, Nature Communications, under the Creative Commons CC BY 4.0 license.

Recent studies have revealed that *C. auris* can adopt an aggregative morphotype during systemic infection, characterized by clustered cell growth and enhanced tissue persistence (**Figure** **3**).^[^ ^113^ ^]^ This phenotype displays preferential tropism for the central nervous system, reduced phagocytic clearance, and partial resistance to host‐derived AMPs such as LL‐37 and PACAP. Genomic analyses of evolved isolates have revealed mutations in regulators of cytokinesis and polarity—including CHS1, BNI1, CAS4, and ACE2—implicating a genetically encoded morphological switch. Interestingly, while aggregation facilitates immune evasion and dissemination, non‐aggregative strains exhibit stronger biofilm formation, greater tissue invasiveness, and higher antifungal tolerance in some settings

### 2.4 C. albicans

*C. albicans* is among the most comprehensively investigated fungal pathogens, particularly with respect to its diverse virulence strategies. These mechanisms include adherence to host and abiotic surfaces, tissue invasion, phenotypic plasticity, secretion of hydrolytic enzymes, biofilm development, nutrient adaptability, fungal thigmotropism, evasion of immune surveillance, and resistance to conventional antifungals, as well as oxidative stress tolerance.^[^ ^114^, ^115^ ^]^ A key virulence trait of *C. albicans* is its morphological versatility, notably its capacity to switch between yeast and filamentous forms (hyphae and pseudohyphae), a transition essential for tissue invasion and systemic dissemination. This morphogenetic shift also promotes the expression of virulence‐associated proteins such as candidalysins, adhesins, and invasins.^[^ ^115^, ^116^, ^117^ ^]^ Interestingly, this dimorphism modulates the host immune response: yeast forms typically elicit Th1 responses, whereas hyphal forms are associated with Th2 immunity.^[^ ^116^, ^118^ ^]^

Following biofilm formation, the shedding of yeast cells enables wider dissemination, supporting systemic spread, immune evasion, and enhanced antifungal resistance.^[^ ^47^, ^119^, ^120^ ^]^ Simultaneously, *C. albicans* produces secreted SAPs and phospholipases (PLB‐A to D), which promote tissue damage and facilitate host invasion.^[^ ^116^, ^117^, ^121^, ^122^ ^]^ Antifungal resistance in *C. albicans* is particularly prevalent among patients receiving long‐term azole prophylaxis, such as those with HIV/AIDS or oropharyngeal candidiasis, although resistance may be transient in some cases.^[^ ^123^, ^124^, ^125^, ^126^ ^]^ Critically, biofilm formation enhances antifungal tolerance—by up to 1000‐fold compared to planktonic cells, primarily by delaying fungicidal activity rather than increasing MIC values.^[^ ^127^ ^]^ This is largely attributed to efflux pump systems, such as the ABC transporters Cdr1 and Cdr2 and the MFS transporter Mdr1, which actively extrude antifungal agents.^[^ ^126^, ^128^, ^129^ ^]^ Although deleting these genes improves fluconazole susceptibility in free‐floating cells, this benefit does not extend to mature biofilms.^[^ ^130^, ^131^ ^]^ The Hsp90 molecular chaperone plays a pivotal role in antifungal resistance by stabilizing key stress‐response regulators like calcineurin and Mkc1, which act within the Pkc1‐MAPK signaling cascade essential for cell wall integrity.^[^ ^130^, ^132^, ^133^, ^134^ ^]^ Beyond azoles, *C. albicans* may also exhibit cross‐resistance to echinocandins, frequently due to mutations in the FKS1 gene, which impair glucan synthase binding and elevate MIC values—contributing to multidrug resistance and nosocomial outbreaks.^[^ ^131^, ^135^, ^136^ ^]^

### 2.5 Current Limitations in Antifungal Treatment

Despite the vast array of antibacterial agents available, the therapeutic options for fungal infections remain severely restricted. Presently, antifungal pharmacotherapy is limited to four primary drug classes—azoles, echinocandins, polyenes, and pyrimidine analogs—each targeting a narrow spectrum of fungal‐specific processes such as ergosterol biosynthesis, nucleic acid replication, and β‐1,3‐glucan synthesis.^[^ ^137^, ^138^, ^139^ ^]^ For instance, flucytosine, a pyrimidine analog, is rarely administered as monotherapy for invasive fungal infections due to its narrow therapeutic window and rapid onset of resistance. A critical challenge across all antifungal classes is toxicity. Amphotericin B, although highly effective, is known for nephrotoxic and hepatotoxic effects, necessitating close monitoring of renal and hepatic function.^[^ ^140^, ^141^ ^]^ Flucytosine can cause bone marrow suppression and hepatotoxicity, especially in patients with pre‐existing renal conditions. Azoles, such as fluconazole, itraconazole, and voriconazole, are generally better tolerated but still pose risks of hepatotoxicity and cytochrome P450‐mediated drug interactions. Voriconazole, in particular, is linked to visual disturbances and photosensitivity. Echinocandins—including caspofungin, micafungin, and anidulafungin—are often regarded as the safest option, though they require intravenous administration and are associated with high costs.^[^ ^142^ ^]^

Each antifungal class presents limitations not only in spectrum and resistance potential but also in the nature of its activity—ranging from fungistatic growth inhibition to fungicidal killing, a distinction with important clinical implications.^[^ ^143^ ^]^ Fungicidal agents actively kill fungal cells, leading to irreversible loss of viability, whereas fungistatic agents merely inhibit growth, relying on host immunity to clear the infection. This distinction is clinically relevant, particularly in immunocompromised patients where fungistatic drugs may fail to achieve clearance.^[^ ^144^ ^]^ For instance, echinocandins are fungicidal against most *Candida* spp. but only fungistatic against *Aspergillus* spp., while azoles are predominantly fungistatic across a broad range of fungi.^[^ ^6^ ^]^

Although Amphotericin B remains active against many clinically relevant fungi—including *Candida* spp., *Aspergillus* spp., and *C. neoformans*—its toxicity restricts its use. Azoles, while effective against *C. albicans*, show limited efficacy against *C. glabrata* and *C. krusei*, with rising resistance reported. Their extensive use, both in healthcare and agriculture, has driven azole resistance, particularly in *A. fumigatus* and multidrug‐resistant *C. auris*.^[^ ^145^, ^146^ ^]^ Echinocandins are fungicidal against *Candida* spp. and fungistatic against *Aspergillus* spp.; however, *Cryptococcus* spp. are inherently resistant due to the absence of β‐1,3‐glucan in their cell wall.^[^ ^147^, ^148^ ^]^ Flucytosine is primarily reserved for combination therapy, such as with Amphotericin B in cryptococcal meningitis, to mitigate resistance emergence and its use as monotherapy is discouraged due to high mutation‐driven resistance.^[^ ^149^ ^]^

The overuse of azoles in both medicine and agriculture has contributed to the emergence of azole‐resistant strains, particularly *A. fumigatus*, now associated with mortality rates up to 88% in specific populations.^[^ ^150^, ^151^, ^152^, ^153^ ^]^ In pathogenic yeasts like *C. albicans, C. auris*, and *C. glabrata*, resistance is often due to mutations in ERG11 or overexpression of efflux pumps (e.g., CDR1, CDR2, MDR1). In filamentous fungi like *A. fumigatus*, mutations in cyp51A and cyp51B are prevalent.^[^ ^154^, ^155^, ^156^ ^]^ Alarmingly, *C. auris* exhibits resistance rates up to 90% for fluconazole, 50% for voriconazole, and 15–30% for Amphotericin B.^[^ ^148^, ^157^ ^]^ Although echinocandin resistance is still uncommon, it is rising in *Candida* spp. due to mutations in FKS1 and FKS2, which impair glucan synthase binding.^[^ ^158^, ^159^ ^]^ In *A. fumigatus*, resistance to echinocandins has also been detected. Additionally, *C. neoformans* is intrinsically resistant to echinocandins due to differences in cell wall structure and enzyme profiles. While Amphotericin B resistance remains rare, isolated cases in *C. albicans* and *C. auris* have been reported, often associated with mutations in ergosterol biosynthesis or responses to oxidative stress.^[^ ^147^, ^160^ ^]^ Despite increasing incidence and resistance rates, the antifungal development pipeline remains limited, with few compounds advancing to late‐stage clinical trials. Innovation is hindered by high development costs, the eukaryotic nature of fungi, and limited druggable targets that differentiate them from human cells.^[^ ^161^ ^]^ New drug classes, including orotomides (e.g., olorofim) and glucosylceramide synthase inhibitors, are under investigation but have not yet achieved broad regulatory approval. The sustained rise in invasive fungal infections—exacerbated by global health crises such as COVID‐19—underscores the need for targeted and scalable antifungal solutions.^[^ ^161^, ^162^ ^]^

Priority fungal pathogens possess a wide array of virulence traits that enable tissue invasion through immune evasion and intrinsic or acquired resistance to antifungal agents.^[^ ^16^, ^163^ ^]^ These include biofilm formation, hyphal transition, secretion of hydrolytic enzymes (e.g., proteases and phospholipases), immune modulation, phenotypic plasticity, cellular aggregation, and stress tolerance.^[^ ^164^, ^165^ ^]^ These mechanisms contribute not only to the establishment of infection but also to reduced susceptibility to conventional antifungal agents—particularly in the context of biofilms, which are often labeled as resistant but typically reflect antifungal tolerance. Unlike true resistance, tolerance does not involve an increase in MIC values, but allows fungal cells to persist under drug exposure by entering a transient, protected state,^[^ ^166^ ^]^ This context underscores an urgent need for novel therapeutic strategies capable of directly targeting and disarming these essential virulence mechanisms to effectively overcome pathogenic fungi.^[^ ^167^ ^]^ Antimicrobial peptides (AMPs) represent a promising class of antifungal agents with multifactorial mechanisms of action that extend beyond membrane disruption, directly interfering with key fungal virulence factors such as biofilm formation and maturation,^[^ ^167^, ^168^ ^]^ by suppressing morphological transitions such as hyphal development, neutralizing secreted hydrolytic enzymes, and restoring immune detection by unmasking β‐glucans or enhancing fungal immunogenicity.^[^ ^169^, ^170^, ^171^ ^]^

## 3 AMPs: A Promising Alternative to Conventional Antifungals

A promising substitute for the current arsenal and the increasing resistance to traditional antifungals would be AMPs, immune response effector molecules made by different species or synthesized. These molecules are short to medium‐length peptides (5–100 amino acids), which have a broad antimicrobial spectrum and also mediate inflammation, proliferation, immunomodulation, and cytokine release.^[^ ^172^, ^173^, ^174^ ^]^ Among other things, these peptides can be α‐helices, β‐sheets, or a combination of both. They can also have a variety of modes of action, including 1) pore creation upon interaction with membranes, 2) effect on cell walls, and 3) suppression of nucleic acids.^[^ ^11^, ^175^, ^176^, ^177^ ^]^

In addition to the previously mentioned mechanisms, peptides with antifungal activity have intricate ways of influencing intracellular structures and functions such as ROS production, mitochondrial dysfunction, apoptosis, autophagy, and cell cycles by interacting with the surface of fungal cells.^[^ ^178^, ^179^ ^]^ Singomycins, iturins, polioxins, echinocandins, leucinostatins, and Skin‐PYY are among the peptide families that have already been described for priority species; nevertheless, the most of them are primarily active against *C. albicans*.^[^ ^177^ ^]^ While some AMPs exhibit fungicidal activity and act through multiple mechanisms that may reduce the likelihood of classical resistance, emerging studies have identified adaptive responses—including cell wall remodeling, protease secretion, and membrane composition changes—that suggest fungi can develop tolerance or even resistance under prolonged exposure. These findings underscore the need for ongoing surveillance and mechanistic studies before broadly assuming a low resistance potential.^[^ ^175^, ^180^ ^]^

### 3.1 Biological Mechanisms of AMPs Against Fungi

Antifungal peptides (AFPs) are cationic molecules with an affinity for cell membranes, whose activity is influenced by properties such as the composition of hydrophobic residues, chain length, and the amphiphilic nature of the sequence, factors that determine their secondary structure and mode of action.^[^ ^181^ ^]^ AFPs constitute a distinct subset of AMPs with selective activity against pathogenic fungi. These molecules, which occur naturally or are chemically optimized, have been designed to enhance structural stability, resist proteolytic degradation, and increase antifungal potency.^[^ ^177^ ^]^ Although few have progressed into late‐stage clinical trials, several AFPs have shown encouraging results in preclinical models, particularly against *Candida* spp. and *Aspergillus* spp., which account for the majority of invasive fungal infections in humans.^[^ ^182^ ^]^ Unlike conventional antifungal agents that typically act on a single molecular target—such as ergosterol biosynthesis or β‐glucan synthesis—AFPs often exert their effects through a combination of mechanisms that include direct membrane disruption, intracellular interference, and inhibition of cell wall assembly. In parallel, many AFPs modulate the host immune response, enhancing antifungal defense pathways. This multifactorial mode of action positions AFPs as attractive candidates for the development of next‐generation antifungal therapeutics, especially in the face of rising resistance to existing drugs.

#### 3.1.1 Membrane Disruption and Permeabilization

The plasma membrane plays a pivotal role in fungal physiology, acting as a selective barrier that controls molecular traffic and mediates interactions with the surrounding environment. The ability of pathogenic fungi to endure environmental stress and evade immune detection is influenced not only by the cell wall but also by the biochemical composition of the plasma membrane, which contributes to membrane fluidity, permeability, and stress response.^[^ ^183^ ^]^ Distinct biochemical features differentiate bacterial from fungal membranes, influencing the susceptibility of each to antimicrobial agents. While bacterial membranes are abundant in phospholipids such as phosphatidylglycerol and cardiolipin, fungal membranes are enriched in sphingolipids, inositol‐containing phospholipids, and ergosterol, the latter functioning analogously to cholesterol in mammalian cells.^[^ ^184^ ^]^ Fluctuations in ergosterol content can significantly affect fungal virulence.^[^ ^185^ ^]^ These molecular distinctions are critical for explaining the varied sensitivity of fungi and bacteria to specific AMPs as they determine the differential binding affinity and membrane disruption patterns observed among microbial taxa.^[^ ^186^ ^]^

Despite the structural similarities between AMPs, such as cationic charge and amphipathicity, specific differences in target membranes, both bacterial and fungal, determine their selectivity and mode of action. Bacterial membranes have a high proportion of anionic lipids, which promote electrostatic attraction of AMPs, facilitating their insertion into the bilayer through hydrophobic regions that induce disorganization, pore formation, and loss of cell integrity.^[^ ^187^ ^]^ Fungal membranes display a distinct lipid composition, including ergosterol and glycosphingolipids such as glucosylceramide (GlcCer), and are accompanied by a robust cell wall composed of chitin and β‐glucans.^[^ ^178^ ^]^

While some AMPs exhibit both antibacterial and antifungal activity, structural and biophysical features strongly influence this specificity. AMPs with β‐sheets stabilized by disulfide bridges tend to be antifungal due to their conformational rigidity, which favors interaction with membrane components, while more flexible and dynamic peptides are generally more effective against bacteria, particularly because they interact with lipopolysaccharides and teichoic acids.^[^ ^187^, ^188^, ^189^ ^]^ In addition, many antifungal AMPs act beyond the membrane, causing oxidative stress, mitochondrial dysfunction, programmed cell death, and autophagy—effects dependent on the peptide entering the cell, such as Psd1 in *C. albicans*.^[^ ^178^ ^]^ Environmental factors such as divalent cations (e.g., Ca^2^⁺, Mg^2^⁺) influence peptide–membrane interactions, sometimes enhancing antifungal activity while diminishing antibacterial efficacy, depending on peptide structure and ionic strength.^[^ ^178^ ^]^ Beyond classical models centered on membrane lysis, it is now recognized that many AFPs act via complex, non‐lytic mechanisms involving intracellular targets and immune modulation.^[^ ^189^ ^]^

Regarding selectivity toward mammalian cells, even though fungal membranes have a lower net surface charge than bacteria,^[^ ^189^, ^190^ ^]^ the presence of anionic phospholipids and ergosterol‐rich lipid microdomains facilitates these interactions. Mammalian membranes, enriched in zwitterionic phospholipids and cholesterol, display lower surface negativity and are thus less permissive to electrostatic interactions with cationic peptides. This physicochemical distinction underlies the selective targeting of fungal over host cells by several AFPs.^[^ ^191^ ^]^ AMPs such as Psd1 demonstrate high selectivity by recognizing GlcCer exclusively in fungi, promoting fungal cell destruction without affecting host cells.^[^ ^192^ ^]^ This lipid specificity is reinforced by residues such as arginine, tryptophan, or diphenylalanine, which amplify the distinction between fungal and mammalian membranes.^[^ ^186^ ^]^ Furthermore, structural modifications that influence peptide conformation and rigidity have been shown to modulate antifungal activity and cytotoxicity toward mammalian cells. For instance, the incorporation of the paramagnetic amino acid TOAC near the *N*‐terminus of the antimicrobial peptide Ctx(Ile^[^ ^21^ ^]^)‐Ha increased its helical content and antifungal activity, but also led to higher hemolytic effects on human erythrocytes. Conversely, TOAC insertion at other positions reduced hemolytic activity, demonstrating that the position of such modifications critically affects membrane interactions and selectivity.^[^ ^193^ ^]^ In addition, structural modifications, such as the incorporation of non‐canonical amino acids and changes at the termini of the peptide chain, have contributed to enhanced selectivity, reduced cytotoxicity, and improved therapeutic efficacy in vivo.^[^ ^194^ ^]^

**Figure 4:**
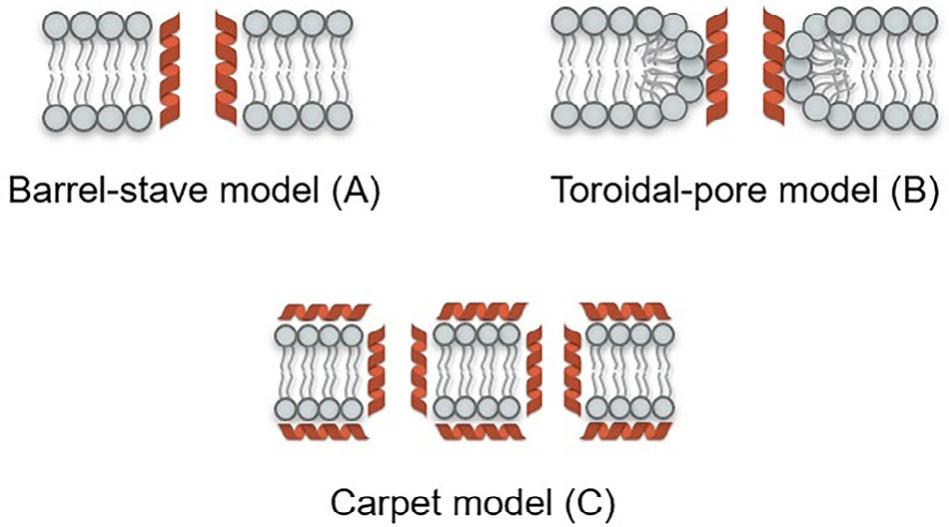
Schematic illustration of the main proposed mechanisms for fungal membrane disruption by antifungal proteins (AFPs): A) Barrel‐Stave Model, characterized by transmembrane insertion of helical structures; B) Toroidal Pore Model, in which bending of the lipid bilayer forms continuous channels with the peptides; and C) Carpet Model, involving surface accumulation on the membrane leading to its disintegration.

Although early studies focused on rapid membrane disruption as the main mechanism of AMPs, it is now known that many peptides act through multifaceted mechanisms, including interaction with the cell wall and intracellular targets, such as mitochondria and vacuoles, resulting in cell death through non‐lytic pathways.^[^ ^189^ ^]^ Initial contact between AFPs and fungal cells is often mediated by cell wall components, including chitin, β‐glucans, and membrane‐associated glycosphingolipids such as GlcCer. These structures, which are absent in bacteria and human cells, contribute to the selectivity of AFPs and facilitate subsequent membrane engagement. Upon binding to the fungal surface, AFPs may destabilize the lipid bilayer, promoting peptide insertion and pore formation. The resulting membrane permeabilization is typically explained by three mechanistic models: Barrel‐Stave, Toroidal Pore, and Carpet (**Figure** **4**).^[^ ^195^ ^]^

In the Barrel‐Stave Model (Figure 4A), peptides initially accumulate on the membrane surface through hydrophobic and electrostatic interactions, potentially favoring ergosterol‐rich domains in fungi. They first align parallel to the membrane surface, then insert perpendicularly into the bilayer, forming pore structures with hydrophobic exteriors and hydrophilic interiors.^[^ ^195^, ^196^ ^]^ Pore formation facilitates ion leakage, ATP efflux, and dissipation of membrane potential, culminating in membrane collapse and cellular dysfunction.^[^ ^197^ ^]^ Experimental support for this model includes the antifungal peptide MAF‐1, derived from *Musca domestica* hemolymph, which has shown efficacy against *C. albicans* by permeabilizing its membrane and impairing vital cellular functions.^[^ ^198^ ^]^ More recently, the human peptide LL‐37, belonging to the cathelicidin family, has exhibited antifungal activity via membrane pore formation and induction of oxidative stress and mitochondrial dysfunction.^[^ ^199^, ^200^, ^201^, ^202^, ^203^, ^204^ ^]^

In the Toroidal Pore Model (Figure 4B), AFPs embed into the lipid bilayer, causing curvature and continuous bending of the membrane, which enables pore formation lined by both peptides and phospholipid head groups.^[^ ^195^, ^205^ ^]^ Consequently, a pore lumen is formed, consisting of peptide molecules interspersed with polar phospholipid heads.^[^ ^205^ ^]^ Unlike the stable and rigid Barrel‐Stave configuration, toroidal pores are dynamic and transient structures in which peptides and lipid headgroups co‐align to form flexible conduits that disrupt the bilayer's hydrophobic–hydrophilic organization. This disruption facilitates new interactions between the lipid tails, allowing for greater flexibility and adaptability in the membrane structure.^[^ ^196^ ^]^ As this process progresses, the peptides cause lipids’ bending around the pore, promoting disorder in the cell membrane and increasing its permeability. As in other pore‐forming models, this permeabilization enables uncontrolled influx of ions and ROS, leading to oxidative stress, membrane depolarization, and ionic imbalance. Following membrane permeabilization, certain peptides may translocate into the cytoplasm and engage intracellular targets.^[^ ^197^, ^205^ ^]^

Studies have shown that peptides that employ the toroidal pore model, such as magainin, lacticin, aurein, and melittin, demonstrate antimicrobial activity by inducing structural and functional dysfunctions in the cell membranes of pathogenic microorganisms.^[^ ^196^, ^206^ ^]^ A similar effect is observed with dermaseptin‐S1 and cecropin A, which influence hyphae formation and cause ion imbalances in *C. albicans*.^[^ ^207^, ^208^ ^]^

In the Carpet Model (Figure 4C), cationic and amphipathic AMPs interact with the surface of the plasma membrane via electrostatic forces, particularly targeting the negatively charged phospholipid head groups. Initially, these peptides align themselves across the membrane surface, generating a uniform layer that mimics the structure of a carpet.^[^ ^197^ ^]^ As more peptides accumulate, a threshold concentration is reached, triggering a destabilizing effect on membrane integrity. At this point, their behavior resembles that of surfactants, causing significant distortion of the phospholipid bilayer. This disruption compromises membrane cohesion, leads to micelle‐like structures, and ultimately culminates in complete membrane rupture.^[^ ^205^ ^]^ A well‐documented example of this mechanism involves dermaseptins, a class of amphipathic peptides secreted by the skin of *Phyllomedusa* frogs. Several peptides employing the Carpet Model, such as dermaseptins, have demonstrated broad‐spectrum antimicrobial activity, although their effectiveness can vary depending on membrane composition, peptide concentration, and environmental conditions.^[^ ^205^ ^]^ In *Candida* species such as *C. albicans* and *C. auris*, dermaseptins impair cell proliferation and hyphal development by downregulating filamentation‐associated genes and inducing oxidative stress, ultimately leading to membrane disruption and cell death.^[^ ^207^, ^209^ ^]^

#### 3.1.2 Intracellular Targeting

A subset of AFPs can bind directly to fungal DNA or RNA, interfering with essential processes such as replication, transcription, and repair. These peptides can associate with nucleic acid backbones or intercalate into grooves, inducing conformational changes that disrupt genomic stability and transcriptional activity.^[^ ^195^, ^205^ ^]^ Although some AFPs can translocate across membranes without causing lysis, their interaction with host nucleic acids or other intracellular components may result in off‐target effects and cytotoxicity. To mitigate these adverse effects while preserving their efficacy against pathogens like *C. albicans* and *A. fumigatus*, strategies such as encapsulation in liposomes or nanoparticles have been developed.^[^ ^205^ ^]^ LL‐37 has been shown to bind and condense nucleic acids, resulting in structural and functional damage to target cells. In *C. albicans* and *A. fumigatus*, LL‐37 was shown to bind and condense nucleic acids, leading to growth inhibition, morphological alterations, and—secondarily—increased membrane permeability, culminating in cell death.^[^ ^210^ ^]^

Another intracellular target of AFPs is the production of essential proteins, as they interfere with ribosomes, blocking the translation of messenger RNA. This leads to the accumulation of misfolded polypeptides and impairs fungal survival.^[^ ^195^ ^]^ Proline‐rich AMPs primarily target bacterial 70S ribosomes and DnaK chaperones; however, analogous strategies are being explored for antifungal applications, although direct inhibition of fungal 80S ribosomes remains less documented. Their effectiveness in inhibiting protein synthesis is enhanced by specific domains associated with Pro, Cys, and acid whey proteins.^[^ ^211^ ^]^

AFPs are also capable of blocking intracellular enzymes that are crucial for fungal metabolism, including polymerases and enzymes involved in protein and lipid synthesis.^[^ ^195^ ^]^ Additionally, these molecules can inhibit enzymes associated with nucleic acid processing, leading to intensified cellular damage.^[^ ^205^ ^]^ A notable example is the peptide CGA‐N9, derived from human chromogranin A, which disrupts the mitochondrial function of *C. tropicalis* by altering metabolic pathways and inducing significant cellular stress.^[^ ^212^ ^]^

Some AFPs promote metabolic damage and structural deregulation by inducing cellular stress and necroptosis through interactions with intracellular organelles, such as the endoplasmic reticulum, and proteins involved in intracellular folding.^[^ ^211^ ^]^ CGA‐N9, for example, induces membrane depolarization, cytosolic calcium overload, mitochondrial ROS accumulation, and release of cytochrome c, ultimately triggering apoptotic pathways including chromatin condensation and DNA fragmentation.^[^ ^212^ ^]^

#### 3.1.3 Inhibition of the Biosynthesis of Cell Wall Components

The fungal cell wall, composed primarily of chitin, mannans, glycoproteins, and glucans, serves as a protective interface against environmental stressors while preserving cellular homeostasis and osmotic balance. The biosynthesis of these structural components relies on key enzymatic pathways, notably chitin synthase and β‐1,3‐glucan synthase. The latter is specifically targeted by echinocandins. These agents act by blocking β‐1,3‐glucan synthesis, a process that compromises the structural integrity of the cell wall, triggers cell stress responses, and can ultimately lead to fungal cell death.^[^ ^197^, ^205^ ^]^ Among these polysaccharides, β‐glucan is the most abundant, playing a dual role in providing mechanical strength and engaging in host immune recognition via receptors such as dectin‐1. Echinocandin antifungals, including caspofungin, micafungin, and anidulafungin, selectively target β‐1,3‐glucan biosynthesis and have proven effective against a wide spectrum of *Candida* spp., including those exhibiting antifungal resistance.^[^ ^213^, ^214^ ^]^

In parallel, nikkomycin Z, a chitin synthase inhibitor, has demonstrated antifungal activity against multiple pathogenic species such as *Blastomyces dermatitidis, Coccidioides immitis*, and several *Candida* strains. It has also been shown to enhance the efficacy of echinocandins and azoles, particularly in disrupting biofilms produced by *C. albicans* and *Candida parapsilosis*.^[^ ^215^ ^]^ Furthermore, nanoparticle‐based delivery systems loaded with nikkomycin Z have improved its efficacy against *Aspergillus* species, offering controlled release and reduced toxicity. A notable synergistic interaction has been reported between nikkomycin Z and fluconazole, facilitating inhibition of germ tube formation in *C. albicans* and highlighting its potential in clinical applications.^[^ ^216^, ^217^ ^]^

Additionally, mannan, a major polysaccharide localized in the outermost layer of the fungal wall, plays crucial roles in adhesion, virulence, and biofilm development. Secondary metabolites such as pradimycins and benanomycins have been identified as mannan‐targeting agents, exhibiting moderate antifungal effects in vitro against *Candida* sp*., Aspergillus* sp., and *C. neoformans*.^[^ ^205^ ^]^ These compounds have also shown in vivo effectiveness, with Pradimycin A and Benanomycin A specifically recognizing D‐mannose in the presence of calcium, initiating cell death through apoptosis and ROS production.

#### 3.1.4 Immune System Modulation

Although often used interchangeably, the terms “immunostimulation” and “immunomodulation” refer to distinct processes. Immunostimulation describes the enhancement of immune responses—such as increased cytokine production or immune cell activation—whereas immunomodulation encompasses broader regulatory effects, which may include both stimulation and suppression, depending on the physiological context.^[^ ^218^, ^219^, ^220^ ^]^ AFPs represent a class of bioactive molecules that not only exert direct antimicrobial effects but also modulate both innate and adaptive immune responses. According to Zhang et al.,^[^ ^182^ ^]^ these peptides recruit macrophages and dendritic cells, enhancing pathogen clearance and promoting immune cell differentiation. Furthermore, they can regulate the production of cytokines such as TNF‐α, IL‐1, and IL‐6, as well as stimulate the differentiation of T cells into the Th17 phenotype, which is fundamental for mucosal immunity.

Certain cationic peptides exert immunostimulatory effects by promoting chemotaxis, wound healing, and lymphocyte activation. They have been reported to enhance tissue regeneration and selectively influence cytokine production, boosting IL‐6–mediated inflammatory responses while also inducing IL‐10 to support immune resolution and tissue repair. Among them, LL‐37 stands out for its ability to regulate specific cellular responses and play a crucial role in defending against infections and chronic inflammation.^[^ ^181^, ^221^ ^]^ IDR‐1018 modulates balanced cytokine responses and restores macrophage plasticity, enhancing resolution of inflammation. The bacteriocin EntV has shown effectiveness in preventing the formation of *C. albicans* biofilms and hyphae, thereby reducing fungal virulence while promoting anti‐inflammatory actions.^[^ ^222^ ^]^ Hepcidin, although not a classical AFP, contributes to antifungal defense by regulating iron availability and indirectly inducing oxidative stress in fungal pathogens.^[^ ^223^ ^]^ Beta‐defensins exert both direct antifungal effects and immunomodulation, while α‐MSH, a neuropeptide with regulatory properties, attenuates inflammation and modulates neutrophil chemotaxis.^[^ ^205^ ^]^

Overall, antifungal peptides occupy a unique position at the interface between antimicrobial defense and immune regulation. Beyond their direct fungicidal activity, many AFPs actively shape the host response—modulating cytokine production, influencing immune cell recruitment, and contributing to tissue repair. This dual functionality challenges the conventional view of antimicrobial agents as purely pathogen‐targeted and opens new therapeutic avenues. Still, their immunological effects are context‐dependent and may vary with infection stage, tissue environment, or host status. A deeper understanding of these dynamics is crucial to optimize AFP‐based therapies, ensuring that immune modulation enhances, rather than complicates, clinical outcomes.

### 3.2 Structure‐Activity Relationship in Antifungal AMPs

The concept of SAR focuses on identifying the link between a molecule's structure and its biological function. In the context of antifungal AMPs, SAR is essential for understanding which structural features are responsible for antimicrobial efficacy. Defining these properties is key for elucidating mechanisms of action and for the rational design of novel AFPs aimed at enhancing antifungal potency.^[^ ^224^ ^]^

#### 3.2.1 Key Structural Features Influencing Antifungal Activity

The ability of these peptides to interact with the fungal membrane depends on chemical characteristics such as the distribution and nature of amino acid residues. Length, for example, is an important parameter; most AFPs have between 11 and 40 residues, with 7 to 8 amino acids being sufficient for the formation of functional amphipathic structures in AMPs, although peptides with fewer than 20 residues have limited effectiveness in forming transmembrane structures in fungal membranes.^[^ ^177^ ^]^

The presence of positively charged amino acids, such as arginine and lysine, is central to the antifungal activity of AFPs, as these residues enable electrostatic interactions with the anionic lipids of the fungal membrane, such as phosphatidylserine, phosphatidylglycerol, phosphatidylinositol phosphates, phosphatidic acid, and glucosylceramides. This attraction favors selective anchoring to the bilayer, promoting the recognition of fungal targets to the detriment of host cells, whose membranes contain mainly neutral lipids.^[^ ^178^, ^225^, ^226^ ^]^ These basic residues are organized into structures known as “cationic claws,” which specifically recognize negatively charged lipids, ensuring the selectivity and effectiveness of the antifungal action.^[^ ^178^ ^]^ Simultaneously, aromatic hydrophobic residues, such as tryptophan, phenylalanine, and diphenylalanine, participate in the insertion of the peptide into the apolar region of the bilayer, promoting destabilization and increased membrane permeability.^[^ ^205^, ^225^, ^226^ ^]^

The positioning of tryptophan at the interface between polar heads and hydrophobic tails favors membrane fluidity and disruption of local lipid organization. Phenylalanine, especially in contexts such as the diphenylalanine motif, contributes to the stabilization of hydrophobic interaction and favors the oligomerization of peptides within the membrane. This reorganization allows the formation of transmembrane channels or pores, in which aromatic residues act as anchors and organizers of amphipathic structures. π‐cationic interactions between aromatic residues and lipid hydrocarbon chains reinforce this process. Ergosterol, a specific component of the fungal membrane, also acts as a functional target by interacting with the aromatic and aliphatic residues of AFPs, contributing to the formation and stabilization of pores and subsequent cellular events, such as loss of transmembrane potential and lysis.^[^ ^225^ ^]^

Experimental data reinforce the functional role of these residues. The replacement of lysine with arginine in peptides derived from the antibiotic K10S, active against *C. albicans*, promoted greater hydrogen bond formation capacity, stabilizing the secondary structure and strengthening interactions with the fungal membrane. The introduction of hydrophobic residues such as isoleucine intensified the insertion of the peptide into the lipid bilayer, increasing the membranolytic effect and inducing cell death. Structural changes involving uncharged polar residues, such as serine and threonine, also impacted the conformation of the peptides, favoring the formation of α‐helices and β‐sheets, structures that modulate stability and interaction with lipids.^[^ ^225^ ^]^ Furthermore, functional examples include the plant defensin MtDef4, whose arginine‐rich RGFRRR motif promotes binding to phosphatidic acid, facilitating its entry into the fungal cell. The NaD1 peptide acts through lysine and arginine residues that interact with PI(4,5)P2, forming oligomeric complexes. Defensin NsD7 forms a helical complex with PA mediated by the same residues, demonstrating how specific amino acids determine antifungal activity by structuring functionally active oligomers.^[^ ^178^ ^]^

**Figure 5:**
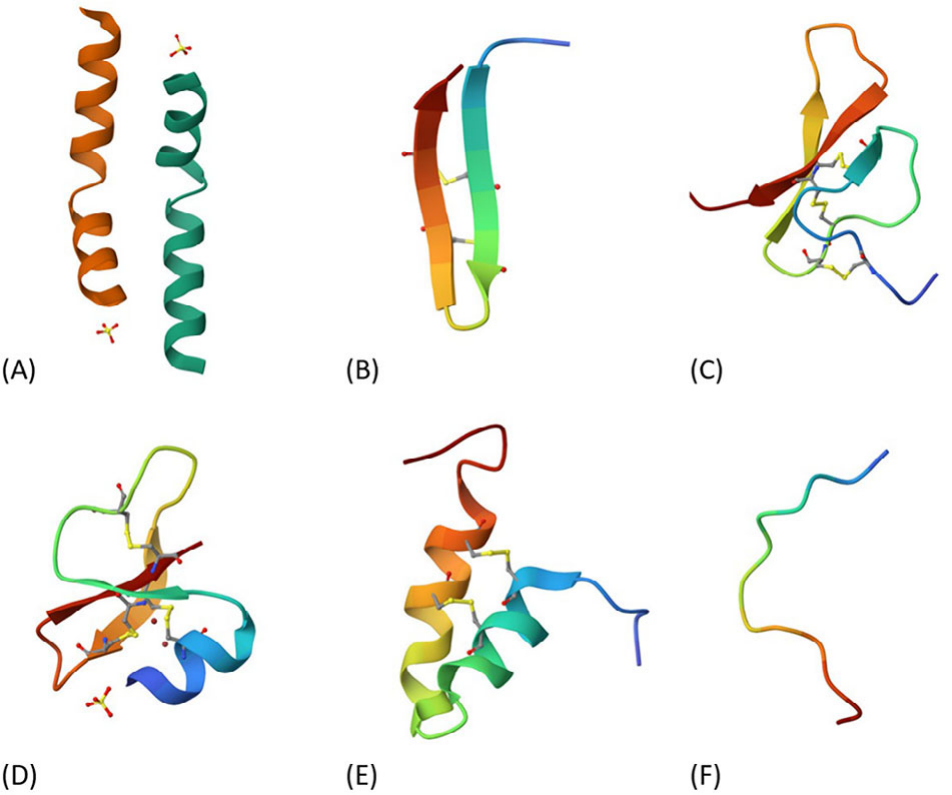
Distinct structural organizations of antifungal peptides, reproduced with permission.^[^ ^225^ ^]^ Copyright 2016, Taylor and Francis. A) α‐helix; B) β‐hairpin; C) Cystine‐knot; D) CSαβ; E) α‐hairpinin; F) extended boat‐shaped structure. Protein Data Bank (PDB) codes: 2MLT, 1PG1, 2DCV, 1IJV, 2L2R, and 1G89, respectively.

AFPs are mainly grouped into two types: helical peptides (α‐helix and 3_10_‐helix), which often exhibit random structures in aqueous solutions but adopt defined conformations in membrane‐like environments; and β‐structured peptides (sheets, hairpins, and barrels), which feature disulfide bridges that stabilize and rigidify their structures. A common structural motif is the cystine‐knot, where three disulfide bridges stabilize antiparallel β‐sheets. Many AMPs, including most defensins, have a cysteine‐stabilized α/β (CSα/β) motif, comprising an antiparallel β‐sheet tethered to an α‐helix by disulfide bonds. Some plant AMPs present the α‐hairpinin motif, characterized by antiparallel α‐helices also stabilized by disulfide bridges. These disulfide bonds confer enhanced resistance to enzymatic, chemical, and thermal degradation (**Figure** **5**).^[^ ^225^ ^]^

Among these, α‐helical AMPs are the most extensively studied due to their effectiveness in forming pores in target membranes. This ability facilitates efficient interaction with the lipid components of the membrane, thereby increasing toxicity against pathogenic organisms while maintaining low toxicity to host cells.^[^ ^227^ ^]^ In contrast, β‐sheet peptides, which are stabilized by disulfide bonds, offer significant efficacy and functional specificity. Meanwhile, αβ AMPs combine the features of both α‐helices and β‐sheets, providing structural versatility and enabling dynamic interactions with cellular targets.^[^ ^228^ ^]^ Peptides that adopt an α‐helix conformation exhibit enhanced efficacy in penetrating cell membranes and inducing damage that ultimately leads to cell death.^[^ ^195^ ^]^ This was further confirmed by Yin et al.,^[^ ^229^ ^]^ who demonstrated that peptides with a predominant α‐helical structure, such as AFP‐13, are particularly effective against fungi. AFP‐13 creates pores in fungal membranes, resulting in cell lysis, with MICs below 10 µg mL^−1^. The Hstn 5 peptide effectively interacts with the membrane of *C. albicans*, adopting an α‐helical structure, resulting in cell death by generating ROS.^[^ ^230^ ^]^

Additionally, studies using membrane models have shown that the Mastoparan B peptide also adopts a helical conformation that enhances its antimicrobial potency, demonstrating the critical role of this structural alteration in its ability to disrupt cell membranes.^[^ ^231^ ^]^ During this mechanism, careful control of amphipathicity is essential, as an excessively high hydrophobic component can increase toxicity to host cells without providing significant improvements in antimicrobial efficacy. Additionally, pathogenic fungi have developed adaptive mechanisms, such as alterations in membrane lipid composition, to diminish the effectiveness of AFPs. This underscores the importance of understanding and leveraging this property to enhance the design of therapeutic strategies.^[^ ^195^, ^227^, ^228^ ^]^

Recent research underscores the practical application of amphipathicity in AFPs for combating resistant fungi. Song et al.^[^ ^232^ ^]^ demonstrated that modifications to the GW4 peptide, including the introduction of hydrophobic residues and Gly, enhanced its efficacy against *C. albicans* by promoting the formation of helical structures. Similarly, Yin et al.^[^ ^229^ ^]^ reported that the AFP‐13 peptide, with an optimized amphipathic structure, exhibited a minimum inhibitory concentration (MIC) of less than 10 µg mL^−1^ against *C. albicans* and *C. tropicalis*. Another notable example is Hstn 5, a human salivary peptide, whose amphipathic organization underpins its strong fungicidal activity against various species.^[^ ^233^ ^]^

The electrical charge of AFPs is a crucial characteristic influencing their interaction with fungal cell membranes, which typically possess a negative charge. AFPs, with charge values ranging from +16 (cationic) to ‐6 (anionic, rare), maximize antifungal activity through electrostatic interactions. Research indicates that an average charge of ≈+6 is optimal for facilitating binding to fungal membrane components, thereby promoting destabilization or rupture of the membrane.^[^ ^228^ ^]^ However, maintaining a careful balance is essential, as excessively high charges may reduce selectivity, while very low charges can increase toxicity to host cells or diminish antifungal efficacy Zh 176 demonstrated that peptides with optimized positive charges, such as magainin, exhibit enhanced efficacy when interacting with negatively charged fungal membranes. Research has shown that the balance of electrical charge is essential for antifungal efficacy. The AFP‐13 peptide showed high activity against species such as *C. albicans* and *C. tropicalis*, due to their optimized positive charge.^[^ ^229^ ^]^ In addition, peptides that combine positive charges with an amphipathic structure demonstrate enhanced efficacy against fluconazole‐resistant fungi.^[^ ^227^ ^]^

#### 3.2.2 Case Studies and Structural Modifications

Modifications of amino acid sequences to increase membrane permeation efficiency, enhancing antifungal activity and minimizing harmful interaction with human cells, contributes to the safe and effective development of AFPs.^[^ ^234^ ^]^ The study by Tancer et al.^[^ ^235^ ^]^ highlights the introduction of myristic acid into peptides, such as AW9‐Ma, which increases efficacy against *C. neoformans*. This modification results in a lower MIC, reaching 64 mg/mL and facilitating the action of antifungals such as caspofungin, by exposing phosphatidylserine on the cell surface of the fungus. The modification of Lys to His residues, as reported by Lacorte Singulani et al.,^[^ ^234^ ^]^ enhanced the electrostatic interaction between the MH58911‐NH_2_ derivative and fungal membranes. This change resulted in improved efficacy against *C. neoformans* biofilms while simultaneously minimizing toxicity to human cells. In addition, the GW4 peptide, which was modified to include glycine at the *N*‐terminus and hydrophobic residues at the *C*‐terminus, demonstrated notable antifungal activity by effectively inhibiting *C. albicans* biofilms. This mechanism was linked to the promotion of apoptosis via pore formation and an increase in ROS levels, as discussed in the research by Song et al.,^[^ ^232^ ^]^ The P113 variant, an amidated derivative of Hstn 5, exhibited a significantly enhanced antifungal efficacy, achieving twice the activity of its original counterpart without necessitating helical conformations, as reported by Sharma et al.,^[^ ^233^ ^]^ Similarly, the K10S peptide was engineered through specific amino acid substitutions to optimize the balance between hydrophobicity and basicity, which led to an increase in its Candidacidal activity compared to the parent peptide.^[^ ^236^ ^]^

In the context of AFPs development, structural modifications can significantly enhance antifungal specificity. For instance, the inclusion of specific residues such as arginine, tryptophan, and diphenylalanine has been linked to improved efficacy. The peptide DipR5 demonstrated promising antimicrobial activities, with MICs ranging from 1.6 to 6.6 µM against *C. parapsilosis* and *A. fumigatus*.^[^ ^186^ ^]^ Furthermore, the analyses conducted by Wu et al.^[^ ^227^ ^]^ emphasize the critical role of point mutations and alterations in electrical charge in modulating the biological activity of AMPs. These findings support the notion that such modifications are not just beneficial but essential for the design of more effective antifungal therapies. Collectively, these studies highlight the importance of peptide engineering as a promising strategy to enhance the antifungal properties of AMPs. Structural modifications in AFPs have shown complementary effects in reducing toxicity while maintaining antimicrobial efficacy.

**Figure 6:**
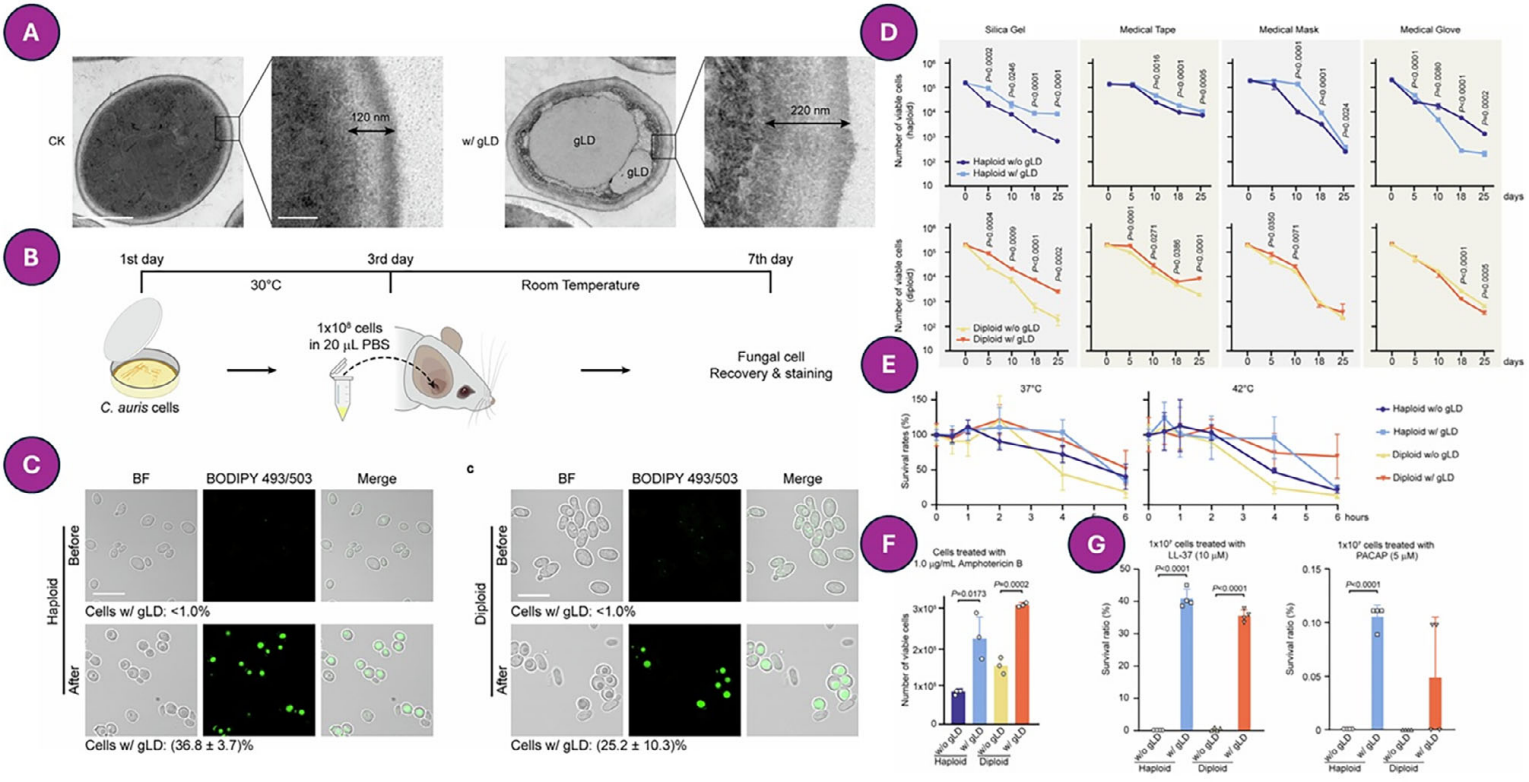
Giant lipid droplets (gLDs) enhance stress resilience and antifungal tolerance in *Candida auris*. A) TEM images show gLD‐containing cells with electron‐dense inclusions, absent in control (CK) cells. B) Experimental design: cultures at 30 °C were injected subcutaneously into mice and recovered at day 7. C) BODIPY staining shows increased gLD‐positive cells post‐recovery (haploid: 36.8 ± 3.7%; diploid: 25.2 ± 10.3%). D) gLD‐positive cells exhibit prolonged viability on abiotic surfaces. E) Enhanced survival under thermal stress (37–42 °C). F) Amphotericin B (1 µg mL^−1^) exposure results in higher survival of gLD‐positive cells. G) gLDs confer increased resistance to LL‐37 and PACAP peptides. Data represent means±SD. Reproduced with permission.^[^ ^237^ ^]^ Copyright 2025, Nature.

Strategies such as the addition of functional groups at the *C*‐terminal of peptides have been effective in decreasing unwanted interactions with human cells, making AMPs more selective and safer.^[^ ^234^ ^]^ Similarly, the conversion of L‐amino acids to D‐amino acids improved the toxicity‐to‐efficacy ratio, with lower hemolytic toxicity observed compared to the original anoplin peptide, while preserving antimicrobial activity.^[^ ^227^ ^]^ The structural optimization of AFPs highlights a broader issue: pathogens such as *C. auris* do not remain static targets. Recent findings show that *C. auris* can form giant lipid droplets, a metabolic shift that confers resistance not only to amphotericin B, but also to host‐derived peptides like LL‐37 and PACAP. These cells show altered chitin deposition and persist on abiotic surfaces, suggesting that stress‐induced lipid remodeling plays a central role in survival and virulence. Unlike classical morphotype switching, this phenotype reflects a deeper metabolic flexibility that complicates therapeutic design, especially in settings with high antifungal pressure (**Figure** **6**).^[^ ^237^ ^]^

Further refinements in antifungal peptide engineering have underscored the importance of precise structural modifications to balance potency and safety. For instance, substitution of lysine with histidine in MK58911‐NH_2_ yielded the derivative MH58911‐NH_2_, which exhibited a fourfold increase in the IC_50_ value (250µg/mL vs. 62.5µg/mL), substantially reducing macrophage cytotoxicity while maintaining antifungal efficacy.^[^ ^234^ ^]^ Similarly, rational introduction of polar residues such as serine and threonine into the K10S backbone (yielding K10S‐TT) enhanced host cell compatibility—preserving >80% viability at antimicrobial doses—by minimizing nonspecific interactions with mammalian membranes.^[^ ^236^ ^]^ Notably, cyclic derivatives such as DipR5 revealed dose‐dependent cytotoxicity in CCRF‐CEM cells, with pronounced effects at 25 µm (84% reduction in viability after 72 h), yet remained selective at lower concentrations (≤10 µm) with negligible effects on noncancerous HEK‐293 cells.^[^ ^186^ ^]^

#### 3.2.3 Peptidomimetics, Cyclization, and PEGylation as Optimization Strategies

The growing resistance of fungal pathogens to azoles, echinocandins, and polyenes—driven by target mutations, efflux pump overexpression, and cell wall remodeling—has exposed the limitations of the current antifungal arsenal.^[^ ^153^, ^183^, ^205^ ^]^ Peptidomimetics, cyclic peptides, and PEGylated derivatives represent complementary strategies to optimize AFPs by improving their stability, pharmacokinetic profile, and target specificity.^[^ ^238^, ^239^, ^240^ ^]^ These chemical modifications enhance resistance to enzymatic degradation, prolong circulation time, and, in some cases, enable synergistic interactions with conventional antifungals by facilitating membrane targeting or intracellular delivery.^[^ ^153^ ^]^

PEGylation has emerged as a widely used strategy to extend peptide half‐life and improve solubility, yet its application in AFPs reveals a more complex interplay between structure and function. By shielding charged and hydrophobic residues, PEG chains can interfere with key interactions between peptides and the fungal membrane—particularly in molecules whose mechanism of action depends on electrostatic attraction and lipid insertion.^[^ ^241^ ^]^ This effect has been exemplified in PEG‐mimicking systems such as the poly(sarcosine)‐functionalized HDP analogs reported by Xie et al.,^[^ ^242^ ^]^ where impaired membrane disruption was accompanied by altered peptide conformation and reduced interaction with ergosterol‐rich fungal membranes. Interestingly, while antifungal potency diminished in some derivatives, the immunomodulatory effects—such as cytokine induction and recruitment of innate immune cells—remained functionally active. These findings open a new line of inquiry into decoupling direct fungicidal activity from host‐directed immune modulation, providing a rationale for designing AFPs with dual or selective therapeutic functions.

These pathogens often reside in difficult‐to‐access niches—biofilms, necrotic tissue, or sites of immunosuppression—where dense extracellular matrices and low perfusion can impede drug delivery.^[^ ^243^ ^]^ While PEGylation enhances pharmacokinetics, it may paradoxically reduce tissue penetration or prevent access to fungal‐specific targets such as β‐glucans or GPI‐anchored proteins.^[^ ^241^ ^]^ This is particularly problematic for peptides that rely on membrane engagement or receptor‐mediated entry. Recent innovations—like cleavable PEG linkers activated by fungal proteases—aim to resolve this trade‐off, offering a controlled release of active peptides precisely where they are needed most.^[^ ^244^ ^]^ As the field moves toward translational antifungal peptide therapies, PEGylation continues to pose both opportunities and limitations. On one hand, it improves systemic stability and reduces immunogenicity; on the other, it may elicit anti‐PEG antibodies with repeated dosing and complicate immune engagement at the infection site. In the context of mycoses that require prolonged treatment, concerns about PEG accumulation and reduced innate immune stimulation become increasingly relevant.^[^ ^245^ ^]^ Thus, while PEG remains a valuable tool, there is growing interest in next‐generation polymeric modifications—zwitterionic moieties, enzymatically cleavable coatings, or biodegradable stealth platforms—that offer pharmacological advantages without blunting the peptide's biological edge.^[^ ^246^ ^]^

Cyclic peptides, with their rigid ring structure, offer high stability against enzymatic degradation, prolonging their half‐life in biological conditions and reducing unwanted interactions and side effects. Whether natural or synthesized in the laboratory, these peptides hold over 40 FDA approvals for uses such as antibiotics, antifungals, and oncological treatments.^[^ ^238^, ^247^ ^]^ Peptidomimetics, on the other hand, mimic natural peptides but incorporate alterations such as β‐amino acids, improving stability, selectivity, and antimicrobial efficacy against fungi.^[^ ^183^ ^]^ Solid‐Phase Peptide Synthesis (SPPS) is widely used in the synthesis of these compounds, while techniques like macrolactamization and orthogonal protection strategies ensure their formation and functionality, with cyclization orientations chosen based on the desired properties.^[^ ^238^ ^]^

Natural cyclic peptides act on microbial membrane disruption and the biosynthesis of essential components of the fungal cell wall, making the development of resistance more difficult. Among the most studied examples are cationic α‐helical peptides, which not only destroy biofilms but also enhance the immune response and exhibit synergistic effects with conventional antibiotics.^[^ ^248^ ^]^ Other examples include polyoxins and nikkomycins, which inhibit chitin synthase, and cyclothiazomycin, which disrupts chitin crystallization in filamentous fungi. Cyclotides, derived from plants and animals, exhibit high structural stability due to the cyclic cystine knot motif, imparting antimicrobial and antiviral properties.^[^ ^249^ ^]^ However, natural cyclic peptides face limitations that hinder their clinical application, such as high immunogenicity, instability due to proteolytic degradation, and challenges in large‐scale production. To overcome these barriers, synthetic peptides have been developed with structural modifications, including truncation of sequences, introduction of non‐natural amino acids, and specific mutations, ensuring greater stability and antimicrobial efficacy. Strategies such as optimizing biophysical properties and incorporating residues of tryptophan, proline, and histidine have demonstrated increased antimicrobial potency while reducing common adverse effects associated with conventional therapies.^[^ ^249^ ^]^

The use of peptidomimetics has shown promising results in attracting and inhibiting pathogenic organisms, without the adverse side effects often associated with natural AMPs.^[^ ^234^ ^]^ A significant example is the α/β peptides developed with β‐amino acids, which displayed antifungal selectivity up to 52 times greater than that of aurein, an effective natural antifungal peptide against various pathogens, including fungi. By utilizing an iterative Gaussian process regression model, it was possible to predict and validate optimized peptide sequences.^[^ ^238^, ^248^ ^]^ The cyclic structure confers resistance to enzymatic degradation on the peptide, enhancing its half‐life and potential for action. Studies indicate that linear AMPs are rapidly degraded in biological conditions, while cyclic variants demonstrate superior efficacy and prolonged action under similar conditions.^[^ ^182^ ^]^ In example, the formation of cyclic peptides from the loop sequence of Cdc50 significantly contributes to protection against proteolytic degradation, resulting in increased antibacterial activity.^[^ ^235^ ^]^

The synthetic peptide DipR5, composed of Arg, Trp, and Phe, demonstrated high stability, with only 15% degradation in the first hour, and exhibited superior efficacy compared to linear AMPs against fungal pathogens.^[^ ^186^ ^]^ Another example is VL‐2397, a cyclic hexapeptide derived from *Acremonium persicinum*, which is active against *A. fumigatus*, *C. neoformans*, and *Candida glabrata*. Its mechanism, similar to that of ferrichrome, disrupts hyphal elongation by chelating iron. In cases of invasive pulmonary aspergillosis, mice treated with VL‐2397 showed increased survival and reduced pulmonary fungal burden.^[^ ^224^, ^250^ ^]^ Advanced technologies, such as artificial intelligence, have the potential to optimize the development of new AFPs, while combinatory therapies with traditional antifungals can enhance efficacy and reduce resistance.^[^ ^183^ ^]^ A notable example is the peptide AW9‐Ma, which, when combined with caspofungin, was able to reduce the MIC to levels comparable to those of mutant strains, such as cdc50D, known for their increased susceptibility to caspofungin. This study demonstrated that the MIC was reduced to 4 mg mL^−1^ in the presence of the peptide, in contrast to the typical resistance exhibited by strains of *C. neoformans*.^[^ ^251^ ^]^ Another relevant example is the combination of the peptide GW4, optimized with the addition of glycine at the N‐terminus, with fluconazole, which displayed a synergistic effect. This effect was measured using the fractional inhibitory index (FICI), which reached a value of 0.25, indicating that the co‐administration of GW4 significantly enhanced the clinical efficacy of fluconazole, leading to a marked reduction in its MIC.^[^ ^232^ ^]^

Cyclization, D‐amino acid substitution, and inclusion of residues such as tryptophan or 3,3‐diphenylalanine facilitate amphipathic alignment and membrane intercalation, especially in ergosterol‐rich fungal membranes. These modifications not only improve serum stability and reduce mammalian cytotoxicity but also enhance biofilm penetration and disrupt tolerance mechanisms. Synthetic peptides like DipR5 and GW4 exemplify this progress, showing potent activity against *Candida* and *Aspergillus* species and synergistic reductions in MIC when used with caspofungin. As such, AFPs with rational structural designs are gaining traction as next‐generation agents capable of addressing key gaps in the antifungal pipeline.^[^ ^186^, ^249^ ^]^ **Table** **1** summarizes the key differences between cyclization and PEGylation in the context of AMP engineering. While cyclization enhances structural rigidity, protease resistance, and target‐binding affinity, PEGylation improves pharmacokinetic properties, including prolonged circulation time and reduced immunogenicity. Each strategy offers distinct advantages and limitations depending on the therapeutic objective—be it membrane disruption, systemic stabilization, or immune modulation.

**Table 1:** Comparative structural strategies for antifungal peptide optimization: Cyclization versus PEGylation.

Feature	Cyclization	PEGylation	References
Advantages	Enhances protease resistance, conformational rigidity, and target‐binding affinity by stabilizing bioactive structures (e.g., θ‐defensins).	Improves solubility, circulation half‐life, and reduces immunogenicity and renal clearance, though steric shielding may impair target interaction.	[252, 253, 254, 255]
Disadvantages	Increases synthetic complexity and cost, and may restrict conformational flexibility needed for dynamic binding.	May reduce antimicrobial potency via steric hindrance, obscure active sites, and suffers from incomplete biodegradability.	[239, 240, 252, 253]
Common Techniques	Head‐to‐tail peptide bonds, disulfide bridges, or side‐chain‐to‐tail linkages; disulfides offer ease but lower redox stability.	Attachment at N‐terminus or lysine residues using linear or branched PEGs; common in systemic AMP delivery.	[240, 253, 255, 256]
AMP Examples	Cyclic lipopeptides, θ‐defensins, cyclized IDR‐1018 analogs, and cyclotides with cystine knot topology.	PEGylated PG‐1 derivatives (e.g., NPG750), PEG‐LL‐37 analogs, and engineered peptides for mucosal delivery.	[239, 240, 252, 253, 255, 256]
Best suited for	AMPs targeting membrane‐bound or receptor‐specific pathogens requiring structural stability and protease resistance.	Systemically administered AMPs where enhanced bioavailability and immunomodulatory effects outweigh direct membrane disruption.	[252, 253, 254, 255]

## 4 Resistance Mechanisms and AMP‐Based Strategies in High‐Priority and Other Fungi

The limited number of antifungal drug classes and the increasing prevalence of resistance among fungal pathogens listed as critical and high‐priority by the WHO pose serious challenges to current treatment strategies. Resistance mechanisms in these fungi range from mutations in ergosterol biosynthetic genes (ERG6, ERG11, ERG3, HMG1), chromosomal aneuploidies, and the overexpression of efflux pumps, to metabolic reprogramming and biofilm‐mediated tolerance, all of which reduce susceptibility to azoles, amphotericin B, and echinocandins.^[^ ^257^ ^]^ These adaptive changes are not only diverse but often cumulative, undermining monotherapies and shortening the clinical lifespan of existing drugs.

Recent attention has turned to pathways absent in humans but essential for fungal survival as alternative therapeutic targets. One such pathway is trehalose biosynthesis, regulated by trehalose‐6‐phosphate synthase (Tps1) and trehalose‐6‐phosphate phosphatase (Tps2). Genetic disruption of *TPS1* or *TPS2* in *C. albicans, C. neoformans*, and *A. fumigatus* significantly impairs fungal growth, stress adaptation, and virulence in vivo, without affecting mammalian cells.^[^ ^258^ ^]^ *TPS1*‐null mutants in *C. albicans*, for instance, display defective filamentation, reduced biofilm formation, and heightened sensitivity to thermal and osmotic stress. With no homologous pathway in humans, trehalose metabolism offers a therapeutic window with minimal off‐target effects. The recent resolution of Tps1 and Tps2 structures by crystallography and cryo‐EM has opened avenues for structure‐based drug design, enabling high‐throughput virtual screening to identify inhibitors that block active sites or disrupt the Tps protein complex.^[^ ^258^ ^]^ This has been further accelerated by AI‐guided docking approaches and predictive ML platforms.

Importantly, targeting trehalose biosynthesis not only limits fungal stress responses but also sensitizes pathogens to membrane‐perturbing agents, including conventional antifungals and AMPs. Combinatorial regimens involving Tps1 inhibitors and amphotericin B or fluconazole have demonstrated synergistic effects, increasing fungal susceptibility to oxidative damage and cell membrane destabilization. In parallel, other conserved lipid biosynthesis enzymes—such as Erg6, Erg24, Erg2, Ole1, and Elo2—have been validated as essential for fungal viability and virulence. Their low homology to human counterparts and key roles in ergosterol and unsaturated fatty acid synthesis make them attractive antifungal targets.^[^ ^8^ ^]^ Although not yet explored in AMP‐based approaches, these enzymes offer a rational direction for future combination therapies.

Against this backdrop, AMPs have emerged as promising candidates to counter resistance by engaging multiple fungal vulnerabilities simultaneously. Unlike classical antifungals, AMPs act through multifactorial mechanisms, often disrupting membrane integrity, inducing oxidative stress, altering ion homeostasis, or triggering apoptosis. In *C. neoformans*, DvAMP—discovered through cheminformatics‐guided screening of over 3 million sequences—disrupts calcium signaling, promotes ROS accumulation, and impairs mitochondrial function, culminating in DNA damage, capsule shrinkage, and biofilm inhibition both in vitro and in *Galleria mellonella* models.^[^ ^259^, ^260^ ^]^ Mo‐CBP3‐PepII exerts similar antifungal pressure, inhibiting antioxidant enzymes (SOD, APX, CAT), disrupting cytochrome c activity, and interfering with ergosterol biosynthesis by targeting LDH.^[^ ^261^ ^]^ Peptide 26d54 adds to this class by modulating transcriptional programs involved in membrane assembly, apoptosis, and DNA repair, thereby compromising fungal homeostasis under stress conditions.^[^ ^262^ ^]^

*C albicans*, well‐known for its capacity to form resilient biofilms and evade immune detection, has shown susceptibility to several plant‐derived peptides including Mo‐CBP3‐PepI/II/III and RcAlb‐PepI/II/III. These peptides disrupt fungal membranes, bind chitin in the cell wall, and induce ROS overproduction, culminating in cellular disintegration.^[^ ^263^ ^]^ Likewise, antifungal cationic peptides achieve rapid fungicidal activity—typically within 8 h at 2–4 × MIC—by compromising membrane integrity, inhibiting hyphal development, and promoting programmed cell death. The AMP ToAP2, in particular, enhances the activity of fluconazole against clinical isolates from catheters by suppressing genes associated with membrane synthesis and efflux pumps.^[^ ^136^, ^264^ ^]^

In *C. auris*, a highly resistant species with global outbreaks, AMPs have shown efficacy even where azoles and amphotericin B fail. The salivary peptide histatin‐5 kills over 90% of fluconazole‐resistant strains at 7.5 µm by translocating into the cytosol and vacuole, leading to intracellular collapse.^[^ ^265^, ^266^ ^]^ Seven additional human‐derived peptides have exhibited MICs of 3.1–12.5 µg mL^−1^ and complete synergy with caspofungin, suggesting therapeutic promise in monotherapy and combination settings.^[^ ^267^ ^]^ Beyond the critical group, several AMPs have also demonstrated activity against high‐priority or neglected fungal pathogens. In *Histoplasma* spp., mastoparan‐derived peptides such as EMP‐AF‐OR display modest activity (MIC: 128 µg mL^−1^), while analogs like MK58911 exert antibiofilm effects by disrupting extracellular matrix rather than directly affecting cell viability.^[^ ^175^, ^268^ ^]^

In *Mucorales* spp., the plant peptide Zeamatin has shown some efficacy,^[^ ^269^ ^]^ whereas synthetic D‐peptides such as d‐KLAKLAKKLAKLAK‐NH_2_ exhibit potent fungicidal activity through membrane depolarization and mitochondrial apoptosis.^[^ ^269^, ^270^, ^271^ ^]^ *Fusarium* spp., including *F. solani* and *F. oxysporum*, are responsive to AMPs like RW and KW, with RW demonstrating superior efficacy.^[^ ^272^ ^]^ Human defensins, cathelicidins, and fragments like hLF1‐11 have shown moderate MICs (10–160 mg L^−1^) and synergism with amphotericin B and voriconazole, likely due to membrane disruption and immune modulation.^[^ ^273^, ^274^ ^]^ However, despite encouraging data, gaps remain. Eumycetoma‐causing agents such as *Madurella mycetomatis* remain largely uncharacterized in terms of AMP susceptibility, and no mechanisms of action have yet been defined.^[^ ^16^, ^275^ ^]^

Moreover, challenges related to AMP stability, immunogenicity, and targeted delivery continue to limit clinical translation. Nonetheless, by engaging conserved fungal processes and bypassing classical drug targets, AMPs offer an attractive complement to current antifungal therapies—particularly when used in rational combinations that exploit specific metabolic or structural weaknesses.^[^ ^276^ ^]^ A clear distinction must be made between antifungal resistance and tolerance, as they reflect fundamentally different biological processes. Resistance arises from stable genetic alterations—such as point mutations in drug targets or sustained upregulation of efflux transporters—that confer the ability to grow at higher drug concentrations, typically reflected by elevated MIC values.^[^ ^277^ ^]^ Tolerance, in contrast, represents a reversible, non‐genetic adaptation in which a subpopulation of fungal cells survives transient drug exposure despite remaining below MIC thresholds. This phenotype is often driven by stress‐responsive pathways, metabolic quiescence, or biofilm‐associated protective states, and contributes disproportionately to treatment failure in persistent or relapsing infections.^[^ ^277^ ^]^

## 5 AMP Conjugation and Nanotechnology Approaches for Enhanced Delivery

### 5.1 Nanoparticles and Target Delivery

Nanotechnology has emerged as a powerful tool to overcome the pharmacological limitations inherent to both natural and synthetic antimicrobial peptides (AMPs). Through the precise engineering of materials at the nanoscale—typically below 100 nm.^[^ ^278^ ^]^ —this approach enables the design of delivery platforms capable of enhancing peptide bioavailability, shielding against proteolytic degradation, and mitigating off‐target cytotoxicity.^[^ ^279^ ^]^ These limitations, if unaddressed, not only diminish therapeutic potential but may inadvertently promote microbial resistance and the selection of multidrug‐resistant strains due to subtherapeutic exposure.^[^ ^280^ ^]^ Consequently, AMP‐conjugated nanocarriers are designed not merely as passive delivery systems but as multifunctional platforms that enhance therapeutic efficacy, protect peptides from enzymatic degradation, reduce systemic toxicity, and enable site‐specific delivery with controlled release profiles.^[^ ^281^ ^]^ **Table** **2** summarizes key nanoparticle‐based strategies for antifungal AMP delivery, highlighting composition, functional features, and therapeutic outcomes across diverse fungal models.

**Table 2:** Nanoparticle platforms for antifungal peptide delivery: composition, characteristics, and key outcomes.

NP type	Composition	NP characteristics	AMP	Study	Main result	References
Inorganic	Mesoporous Silica	High thermal and chemical stability, biocompatibility	LL‐37	Investigate how properties influence the loading, release, and protection of LL‐37 against degradation by proteases.	Negatively charged particles with high surface area demonstrated a superior ability to incorporate AMPs, protecting them against degradation by infection‐related proteases.	[291]
Lipidic	Lipid NPs	Composed of lipids that form a solid matrix or capsule to encapsulate AMPs.	Various AMPs are being explored as alternatives to conventional antibiotics	The review provides an update on recent advancements in NP systems for AMP delivery, focusing on their pharmaceutical applications.	The findings suggest that the use of NPs can significantly improve the delivery and efficacy of AMPs, addressing challenges such as solubility, stability, and toxicity	[292]
Nanofibers	Titanium dioxide (TiO_2_) and cerium dioxide (CeO_2_)	The combined structure of TiO_2_ and CeO_2_ enhances photocatalytic properties, thereby boosting antifungal activity.	Synthetic AMPs based on pilosulin and ponericin	The nanofibers were evaluated against biofilms of *Candida glabrata*, *C. albicans*, and *Candida krusei*.	TiO_2_‐CeO_2_ nanofibers demonstrated significant inhibition of biofilm development, effectively reducing cell adhesion and proliferation of fungal cells.	[293]
Liposome	Phospholipids	Low cytotoxicity, with cell survival even at higher concentrations	hLF, dhvar4, dhvar5, UBI 18–35 and UBI 29–41	The study aims to explore the role of antifungal peptides as a promising treatment option against azole‐resistant *A. fumigatus*.	The peptides evaluated exhibited dose‐dependent antifungal activity against *A. fumigatus*. Among them, dhvar5 showed the best results, indicating it was particularly effective at inhibiting the growth of the fungus.	[294]
Metallic	Gold NPs	Chemically stable due to the noble nature of gold, making them resistant to oxidation under normal conditions	C7H2 and HuAL1	The study evaluated the antimicrobial activity of the peptide‐conjugated AuNPs against several microorganisms, including *C. albicans*.	The results showed that the AuNPs conjugated with the HuAL1 peptide exhibited the highest antimicrobial activity against *C. albicans*, achieving a maximum inhibition of microbial growth.	[295]
Metallic	Gold NPs	The NPs exhibit stability across various pH levels	Ampicillin	The study investigates the interaction of AMP‐Gold particles with bacterial cells, focusing on their efficacy against both sensitive and resistant ampicillin microorganisms.	The efficacy of these NPs increased up to sixteen‐fold against ampicillin‐sensitive bacteria and four‐fold against ampicillin‐resistant *C. Albicans and E. coli*.	[296]
Metallic	Gold NPs	They exhibit antimicrobial properties and can enhance the stability of AMPs against protease degradation	Indolicidin	Investigates the conjugation of indolicidin to AuNPs, which showed significantly higher activity against *C. albicans*.	The studies highlighted that the combination of AMPs with NPs not only enhances antimicrobial activity but also provides stability and protection against degradation.	[297]
Nanoemulsion	Oil, water, and surfactants	More stable than macroemulsions because the small droplet size reduces phase separation	Nisin, pediocin, and lactoferrin	The studies focus on the effectiveness of nano‐conjugated AMPs in food preservation, in active packaging applications.	The use of nano‐conjugated AMPs significantly enhances their antimicrobial activity, stability, and delivery, leading to improved food safety and shelf life.	[298]
Polymeric	Chitosan	Protect AMP from degradation and increase its stability	AMP‐17	Develop and evaluate the antifungal efficacy of AMP‐17 encapsulated in chitosan NPs against *C. albicans*.	AMP‐17 encapsulated in chitosan NPs showed significant antifungal effect by causing cell wall and cell membrane disruption	[299]
Polymeric nanofibers	PCL/PLGA	Good mechanical stability, biocompatibility and sustained release of active compounds	LfcinB (21–25)Pal	Evaluate the antimicrobial activity of PCL/PLGA nanofibers incorporating LfcinB (21–25)Pal in the inhibition of polymicrobial biofilms	Nanofibers demonstrated strong antibiofilm and antimicrobial activity, significantly reducing the viability of *C. albicans* and polymicrobial biofilms.	[300]
NP formed by lipoic acid	Lipoic acid (LA) and PEA	The engineered NPs demonstrate biosafety profiles	RWR tripeptide	Develop and assess new strategies to improve the AMPs, focusing on their effectiveness against fungi.	The results indicated that the NPs exhibited excellent antifungal activity against *C. albicans*, with significantly lower MIC values compared to the unmodified LA‐RWR peptide.	[301]

NPs: Nanoparticles, PLGA: Poly(lactic‐co‐glycolic acid), PEG: Polyethylene glycol, PCL: Polycaprolactone; PEA: Phenethylamine.

Various chemical and structural strategies have been implemented to improve AMP stability within biological systems. Rational design of peptide sequences to avoid protease‐sensitive motifs,^[^ ^282^ ^]^ conjugation with protease inhibitors to block enzymatic degradation,^[^ ^283^ ^]^ and the substitution of natural residues with non‐natural or D‐amino acids,^[^ ^284^ ^]^ have all shown efficacy in extending peptide half‐life. However, while such modifications improve biochemical resilience, they may also alter biological activity or receptor interactions. Therefore, embedding AMPs within nanoscale delivery systems—particularly lipidic and polymeric formulations—has emerged as a complementary or alternative strategy to retain peptide functionality while mitigating pharmacokinetic drawbacks.^[^ ^285^ ^]^

Lipid‐based nanocarriers, including liposomes, micelles, solid lipid nanoparticles, and lipid nanocarriers, have demonstrated a strong capacity to encapsulate AMPs, thereby shielding them from environmental degradation and enzymatic attack.^[^ ^286^ ^]^ These systems are not only biocompatible but also allow modulation of release kinetics through adjustments in lipid composition and particle size, offering precise control over pharmacodynamics. Parallel developments in polymeric nanocarriers—particularly those based on FDA‐approved materials such as PLGA—have enabled further advancements in AMP delivery. PLGA‐based nanoparticles and microspheres optimize release profiles through controlled degradation and physicochemical tuning, while minimizing local and systemic toxicity.^[^ ^287^ ^]^ Their biodegradability and low immunogenicity render them suitable for chronic administration and tissue‐specific applications. Beyond classical carriers, emerging nanosystems such as nanofibers and hydrogels have expanded the potential of AMP‐based therapy. These structures support sustained and localized release, with added benefits in wound healing and infection management.^[^ ^288^ ^]^ For instance, liposomes—lipid bilayer vesicles capable of encapsulating both hydrophilic and hydrophobic molecules—are not only protective carriers but also exhibit reduced cytotoxicity, making them attractive for both biomedical and food industry applications.^[^ ^281^, ^289^, ^290^ ^]^

Distinct nanoparticle types confer different advantages when conjugated with AMPs. Metallic nanoparticles, such as silver and gold, have been widely explored due to their inherent antimicrobial activity and capacity to stabilize peptide cargo.^[^ ^302^ ^]^ These systems have demonstrated efficacy in extending the antimicrobial spectrum of AMPs, notably against Escherichia coli resistant to ampicillin and *C. albicans*, by enhancing peptide uptake and promoting ROS‐mediated damage.^[^ ^296^ ^]^ The combination of metallic nanoparticles with AMPs has also been associated with improved selectivity toward multidrug‐resistant strains, while preserving low cytotoxicity profiles—a critical balance for clinical translation. Nanofiber‐based systems represent another frontier. In particular, TiO_2_–CeO_2_ nanofibers have shown potent antifungal activity by inhibiting *Candida* biofilms via photocatalytic disruption of cell walls.^[^ ^293^ ^]^ AMP‐loaded nanofibers composed of chitosan, PCL, or PLGA have further demonstrated antimicrobial and antibiofilm efficacy against *C. albicans* and polymicrobial consortia.^[^ ^299^, ^300^ ^]^ These platforms not only improved peptide solubility and biological stability but also suggest a route for sustained, localized antifungal therapy.

While broad nanoparticle strategies focus on protecting AMPs and improving pharmacokinetics, the next generation of delivery systems emphasizes precision targeting. Functionalization of nanoparticles with biological ligands—such as peptides, antibodies, glycoproteins, or polysaccharides—has been employed to direct AMP‐loaded systems to fungal‐specific surface markers or inflammation‐associated sites.^[^ ^303^, ^304^ ^]^ This targeting minimizes off‐site distribution, concentrates the therapeutic payload at the infection site, and reduces collateral toxicity. The use of peptide ligands such as Histatin 5 has shown promise in targeting fungal receptors, ensuring high local accumulation of AMPs and enhancing efficacy.^[^ ^304^ ^]^

**Figure 7:**
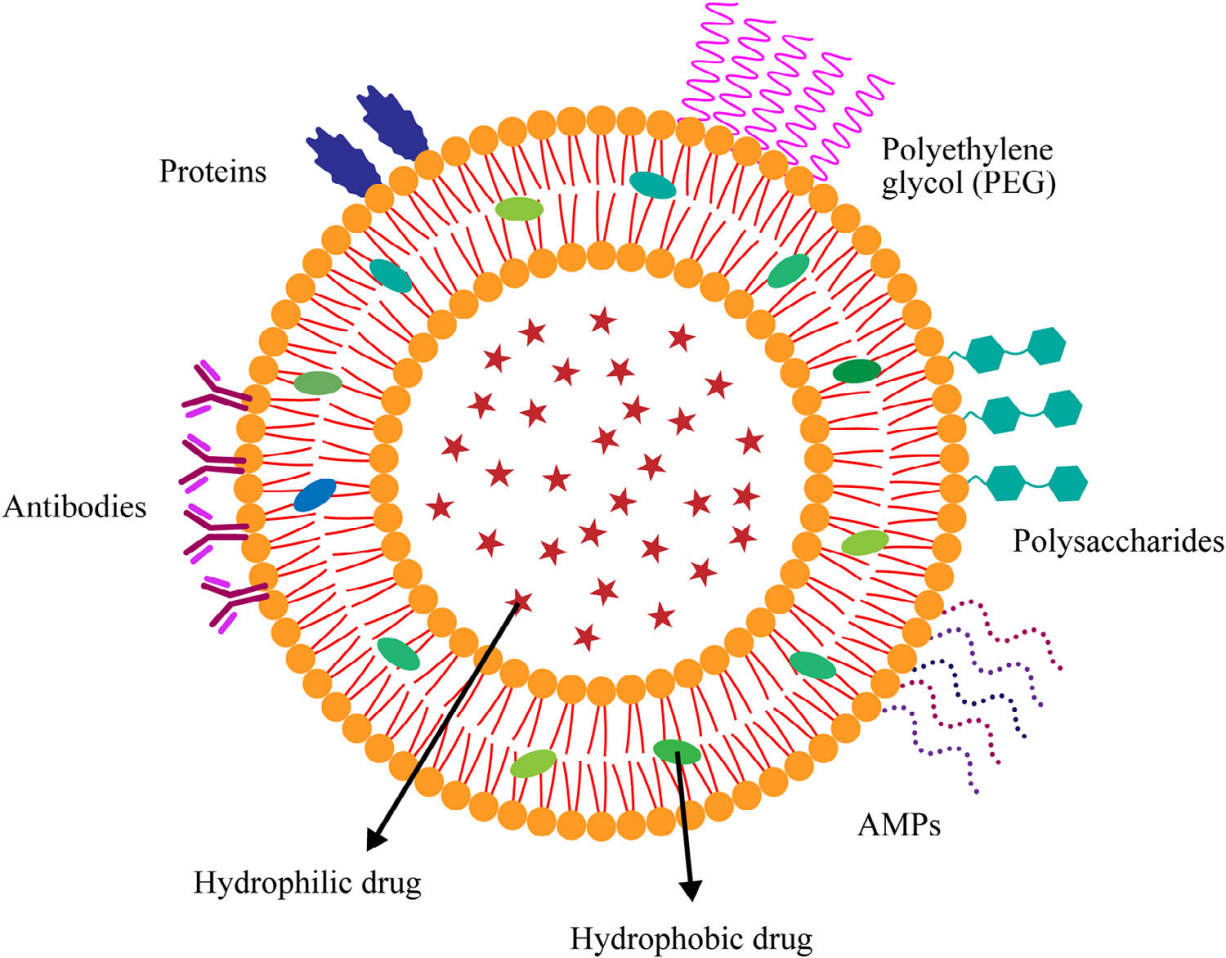
Schematic representation of a surface‐functionalized liposome for targeted drug delivery. The liposomal structure encapsulates both hydrophilic drugs in its aqueous core and hydrophobic drugs within the lipid bilayer. The liposome surface could be functionalized with various biomolecules—including polyethylene glycol (PEG), polysaccharides, antimicrobial peptides (AMPs), antibodies, and proteins—to enhance stability, immune evasion, targeting specificity, and therapeutic efficacy.

Stimuli‐responsive systems add a further layer of control by enabling on‐demand peptide release triggered by environmental cues such as pH, redox state, temperature, or electromagnetic fields. These technologies are particularly useful in treating deep‐seated or refractory infections, where conventional therapies fail to reach therapeutic thresholds. As depicted in **Figure** **7**, surface‐functionalized liposomes represent an archetype of targeted nanodelivery. Such systems may incorporate PEG chains, targeting ligands, or antifungal peptides to improve systemic circulation, evade immune clearance, and ensure focused activity at pathological sites.^[^ ^305^ ^]^ A growing body of experimental evidence supports the therapeutic utility of AMP–nanoparticle systems. The conjugation of AMPs with inorganic materials, notably silver and gold, has been shown to enhance cell wall penetration and generate ROS for rapid microbial killing.^[^ ^306^ ^]^ Functionalization with chitosan—an antimicrobial, mucoadhesive polysaccharide—further augments the bioactivity and retention of AMPs, particularly in mucosal and gastrointestinal applications. Although peptide–polymer conjugates for antifungal therapy remain relatively underexplored, chitosan‐based systems have demonstrated robust performance in antibacterial contexts and in sensitizing antibiotics,^[^ ^307^, ^308^ ^]^ providing a rationale to extend these strategies to fungal pathogens.^[^ ^309^ ^]^

Covalent conjugation techniques such as amide bonding between nanoparticle carboxyl groups and peptide amines, esterification for linkage to polymer chains, and amination reactions for surface grafting are commonly employed to ensure structural integrity and release control.^[^ ^310^ ^]^ Electrostatic interactions, while simpler, are inherently less stable and more susceptible to physiological disruption. Therefore, choosing the appropriate conjugation chemistry remains a pivotal consideration in nanoparticle design. Notably, Jayathilaka et al.^[^ ^309^ ^]^ encapsulated the AMP octominin in chitosan nanoparticles, significantly improving its antifungal efficacy against *C. albicans* while reducing cytotoxicity in HEK 293 cells and demonstrating safety in zebrafish up to 50 µg/mL. The formulation improved biofilm inhibition and eradication, offering a viable route for topical or systemic AMP administration.

Similarly, Formaggio et al.^[^ ^295^ ^]^ showed that AuNPs conjugated with HuAL1 completely inhibited fungal growth at 1.2 mg/mL, whereas dual‐peptide systems showed diminished activity, suggesting that conjugation can alter peptide structure and function. Beyond infectious disease, peptide‐nanoparticle systems have been explored in regenerative medicine. Gomes et al.^[^ ^311^ ^]^ developed C16‐Im‐PP4, a conjugate between an imidazolium‐based ionic liquid and a collagen‐inducing pentapeptide. The hybrid showed antimicrobial activity against *Candida* spp., as well as antioxidant, anti‐inflammatory, and pro‐healing properties in diabetic wound models—accelerating re‐epithelialization, reducing ROS, and enhancing collagen deposition. This underscores the broader therapeutic versatility of AMP conjugates beyond direct antimicrobial action.

Recent studies have also validated the use of manganese ferrite (MnFe_2_O_4_) nanoparticles functionalized with citric acid and conjugated to the AMP Cmp5, resulting in synergistic antifungal activity against *C. albicans* with reduced MIC values compared to peptide alone.^[^ ^312^ ^]^ This not only demonstrates the potential for dose sparing but also reinforces the importance of physicochemical tuning in maximizing therapeutic outcomes. The functionalization of AMPs with polyethylene glycol (PEG) is another widely adopted strategy that enhances systemic circulation, minimizes proteolysis, and reduces immunogenicity and aggregation.^[^ ^313^ ^]^ While PEGylation may attenuate direct antimicrobial activity—particularly in membrane‐active peptides—it often preserves or enhances immunomodulatory effects, offering an alternative mechanism of therapeutic action.^[^ ^314^ ^]^

### 5.2 AMPs Conjugation Strategies

The clinical use of AMPs faces challenges due to their low in vivo stability, toxicity, and adverse immunological interactions. Conjugation refers to the process of chemically linking two molecules together. In the context of drug development, this often involves attaching a drug to a carrier or another molecule to enhance its properties.^[^ ^315^ ^]^ The conjugation of AMPs with other AMPs forms hybrid compounds that are more effective and stable. This strategy employs covalent bonds to preserve peptide functionality and facilitate efficient internalization into pathogens, although challenges remain in optimizing the conjugation conditions.^[^ ^316^ ^]^ Strategies for developing new AMPs include hybridization, structural modifications, and computational tools. Hybridization involves combining AMP fragments to enhance activity and broaden antimicrobial spectra. Structural modifications, such as lipidation and cyclization, improve stability, efficacy, and selectivity.^[^ ^317^ ^]^

The conjugation of AMPs can be achieved SPPS via, linking fluconazole to the N‐terminal amino group of peptides using glutaric acid as a linker and after peptide synthesis, fluconazole is attached in the final step, and the conjugates are purified and characterized. In a recent study, five peptides with antimicrobial and cell‐penetrating potential were selected: TP10‐NH_2_, TP10‐7‐NH_2_, LFcinB(2‐11)‐NH_2_, LFcinB[Nle1,11]‐NH_2_, and HLopt2‐NH_2_. The conjugates showed significant antifungal activity against *C. albicans*, including fluconazole‐resistant strains, but lower efficacy against *Candida glabrata* and *Candida krusei*.^[^ ^318^ ^]^ A noteworthy example is the conjugation of caspofungin derivatives, an antifungal peptide from the echinocandin family, which is effective against *Candida*, *Aspergillus*, and other resistant fungal species by inhibiting the synthesis of β(1,3)‐D‐glucan, an essential component of the fungal cell wall.^[^ ^319^ ^]^

One strategy to enhance the therapeutic potential of AMPs is the chemical modification of the compounds, which can improve stability, selectivity, and antimicrobial activity.^[^ ^320^ ^]^ To overcome challenges such as degradation, toxicity, and high production costs, modifications at the termini, such as acetylation or methylation, increase resistance to proteases and circulating half‐life and the inclusion of non‐standard amino acids reduces proteolytic degradation, preserving or enhancing antimicrobial efficacy.^[^ ^321^ ^]^ The study by Pineda‐Castañeda et al.,^[^ ^322^ ^]^ presented synthesis routes for conjugating AMPs to a resorcinarene core using innovative techniques. Initially, the resorcinarene core was functionalized with alkyne groups through acid‐catalyzed cyclocondensation. Then, the linear peptides LfcinB and Buforin were synthesized via SPPS and functionalized with azide groups at their N‐terminal ends using Fmoc‐azidolysine. Finally, the functionalized peptides were conjugated to the resorcinarene core through copper‐catalyzed azide‐alkyne cycloaddition, resulting in tetravalent conjugates, which were tested against *C. albicans* and *C. auris*.

## 6 Emerging Trends in AMP‐Based Antifungal Therapeutics

### 6.1 High‐Throughput Screening and Design of Synthetic AMPs with Enhanced Antifungal Properties

Synthetic AFPs have been rationally designed to interact specifically with fungal membranes, including host defense peptides and cell‐penetrating peptides (CPPs).^[^ ^323^ ^]^ These peptides typically interact with the fungal cell surface, membrane, and cell wall. Their mode of action involves the formation of amphipathic helical structures (α‐helices, β‐sheets, or mixed motifs) upon membrane association,^[^ ^324^ ^]^ enabling insertion into the lipid bilayer and induction of pore formation through membrane curvature. Fungal membranes consist of sterols, phospholipids, and sphingolipids, while the fungal cell wall is composed of glucans, chitin, and glycosylated proteins, including adhesins and receptors.^[^ ^325^ ^]^

Recent strategies have aimed at improving AMP internalization efficiency by conjugating peptides with lipid‐based formulations. For example, AMPs have been tethered to LBF127 beads, facilitating their insertion into fungal unilamellar vesicles.^[^ ^326^ ^]^ This approach led to the design of a peptide variant, K‐oLBF127, which exhibited improved membrane interaction against *C. neoformans*.^[^ ^327^ ^]^ Other synthetic peptides include IDR‐1018, a cationic AMP with activity against *C. albicans*, known to induce cytokine production during macrophage differentiation and promote immune modulation.^[^ ^328^ ^]^ RQ18, a computationally designed AMP, demonstrated favorable physicochemical properties and selective membrane binding via interaction with ergosterol. RQ18 acts synergistically with amphotericin B to enhance antifungal efficacy.^[^ ^329^ ^]^ Additionally, albumin‐derived peptides such as Alb‐PepI and Rc Alb‐PepII have shown antimicrobial potential with low toxicity. Rc Alb‐PepII demonstrated both bacteriostatic and bactericidal activity against Klebsiella pneumoniae, acting through cell wall disruption rather than membrane permeabilization.^[^ ^330^ ^]^ The synthetic peptide Hulk also exhibited strong antifungal activity against *Fusarium oxysporum* and *Botrytis cinerea*.^[^ ^331^ ^]^ These examples highlight the ongoing efforts to develop synthetic AMPs with improved solubility, extended activity, and scalable production for clinical application.

### 6.2 Peptide Libraries and Computational Approaches for Rational AMP Design

The development of synthetic AMPs has been significantly accelerated by the establishment of comprehensive peptide databases and bioinformatic tools. These include the Collection of Anti‐Microbial Peptides (CAMPR), Database of Antimicrobial Activity and Structure of Peptides, Data Repository of AMPs (DRAMP), and the Antimicrobial Peptide Database.^[^ ^332^ ^]^ These databases classify AMPs based on origin, taxonomy, and antimicrobial activity (bactericidal, antiviral, antifungal, antiparasitic).^[^ ^333^ ^]^ Rational screening approaches in peptide libraries focus on designing AMP sequences based on desired functional traits (e.g., charge, hydrophobicity, helicity), either through natural analogs or modified sequences generated in silico.^[^ ^334^ ^]^ In contrast, non‐rational screening relies on random libraries of small molecules to identify novel bioactive compounds using target‐based or ligand‐based discovery strategies.^[^ ^335^, ^336^ ^]^

Artificial intelligence (AI) encompasses a broad family of algorithms, including machine learning (ML) and its more specialized subset, deep learning (DL). ML typically relies on feature engineering and structured data to build predictive models, whereas DL employs multilayer neural networks that learn directly from raw sequences or structural inputs. In the context of AMP design, ML has proven effective in classification tasks, activity prediction, and toxicity profiling, while DL offers enhanced capabilities for de novo peptide generation and structure–activity mapping—especially against fungal targets. The integration of AI‐driven modeling with curated AMP libraries has improved the prediction of key pharmacological parameters such as selectivity, bioavailability, proteolytic stability, and host cytotoxicity.^[^ ^332^ ^]^ Computational tools have become essential in antifungal peptide discovery, enabling virtual screening, secondary structure prediction, membrane interaction simulations, and docking with fungal proteins or RNA elements.^[^ ^337^, ^338^ ^]^ Recent advances also include generative AI platforms capable of proposing novel AMP scaffolds based on optimized physicochemical filters and learned antifungal signatures.^[^ ^330^, ^335^, ^336^, ^339^ ^]^

**Figure 8:**
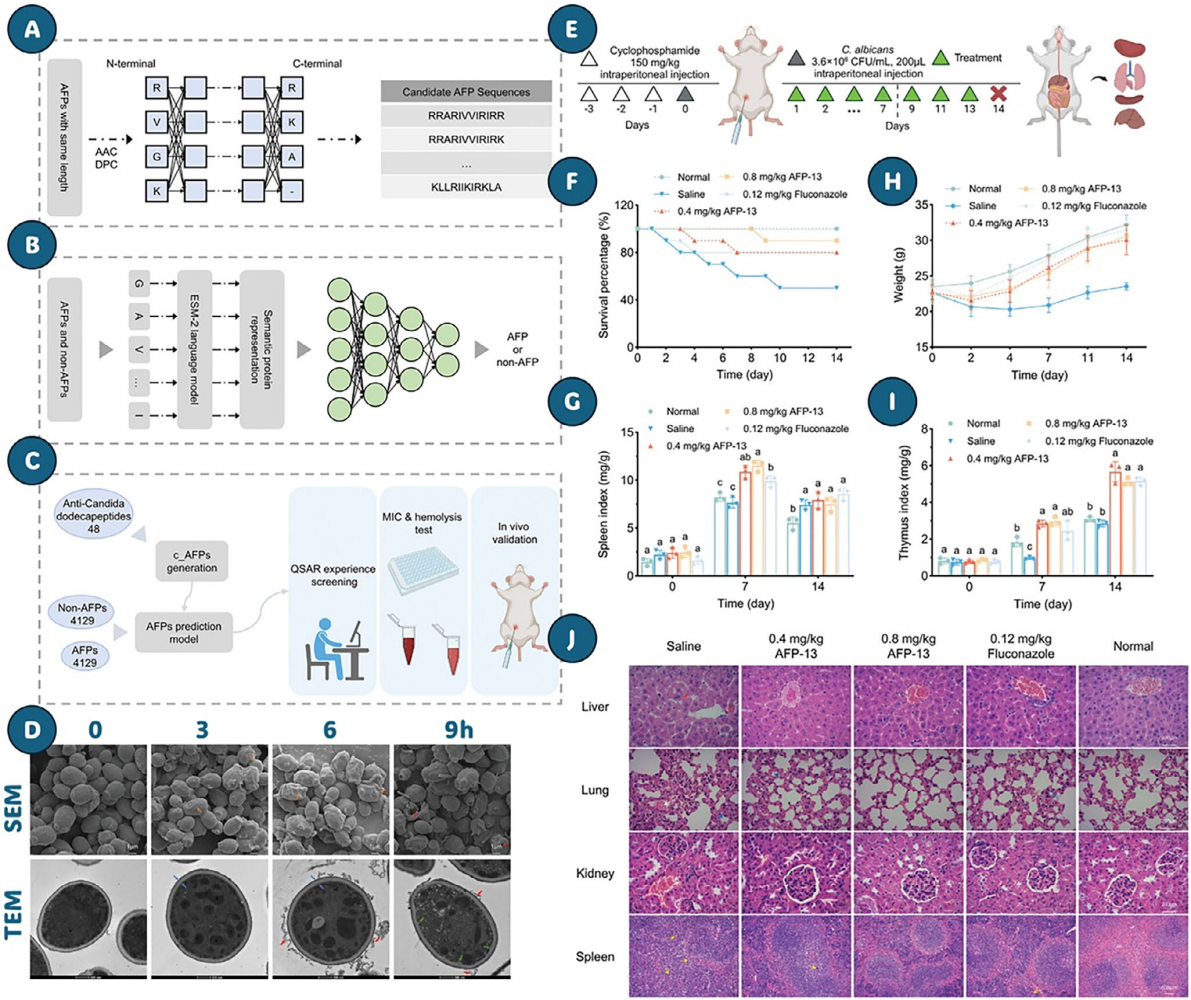
Deep learning‐guided discovery and in vivo validation of AFP‐13 against fluconazole‐resistant *C. albicans*. Overview of the DL‐QSARES workflow: A) Candidate antifungal peptide (AFP) motifs derived from amino acid composition and dodecapeptide fragments; B) deep learning model based on BERT‐MLP architecture to classify AFPs vs. non‐AFPs; C) in silico QSAR filtering and empirical screening pipeline for AFP prioritization. D) SEM and TEM imaging reveal time‐dependent membrane disruption and cytoplasmic leakage in C. albicans treated with AFP‐13. E) Experimental design for a murine model of systemic infection using a fluconazole‐resistant C. albicans strain. F) Survival curve shows >90% survival in mice treated with AFP‐13 (0.8 mg kg^−1^), outperforming fluconazole and saline. G–I) Immunological indicators (spleen and thymus indices) confirm systemic recovery in AFP‐13–treated animals. H) Body weight progression during treatment. J) Histological analysis (H&E staining) of major organs shows preserved tissue integrity in AFP‐13–treated mice, indicating therapeutic efficacy and low toxicity. Reprinted/adapted from Yin et al.,^[^ ^340^ ^]^ Advanced Science, under the Creative Commons CC BY 4.0 license.

The recent study by Yin et al.^[^ ^340^ ^]^ showcases a powerful application of DL in antifungal peptide discovery through the development of the DL‐QSARES platform—a multitask architecture combining Bidirectional Encoder Representations from Transformers with a multilayer perceptron to predict antimicrobial activity, cytotoxicity, and target interaction. This model integrates quantitative structure–activity relationships (QSAR) into a deep learning framework, enabling the de novo generation and screening of AFPs with high precision. Among the 42 DL‐designed candidates, AFP‐13 was selected based on strong in silico predictions, including AlphaFold3 modeling and docking with fungal phospholipids and ergosterol‐binding sites. AFP‐13 demonstrated broad‐spectrum activity, particularly against *C. albicans*, including fluconazole‐resistant strains, with MIC values as low as 3.1 µg mL^−1^. In a murine model of systemic candidiasis (**Figure** **8**), AFP‐13 achieved 90% survival over 14 days, significantly reduced fungal burden in multiple organs, and downregulated inflammatory cytokines (IL‐1β, TNF‐α, IL‐6), indicating both antifungal and immunomodulatory effects. Notably, AFP‐13 retained activity in physiological salt and serum conditions, and showed no hemolytic or cytotoxic effects in mammalian cells, highlighting its pharmacological robustness. These results establish DL‐QSARES as an efficient strategy for accelerating peptide optimization pipelines, reducing experimental burden, and improving translational potential in antifungal drug development.

**Figure 9:**
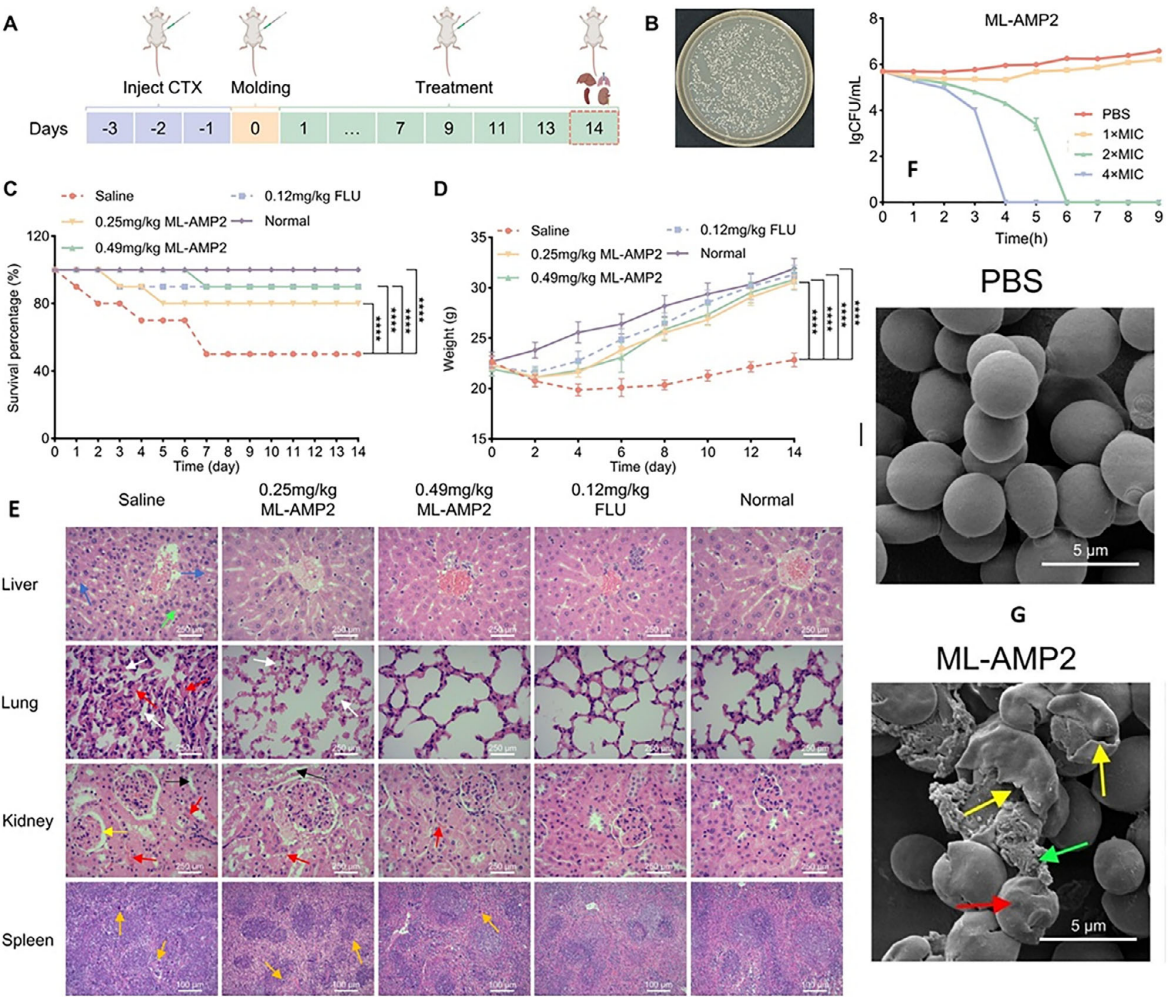
In vivo efficacy and histopathological impact of ML‐AMP2 in a murine model of systemic candidiasis. A) Experimental timeline: mice were immunosuppressed with cyclophosphamide (CTX), infected with *C. albicans*, and treated with ML‐AMP2 or controls for 14 days. B) Fungal colony grown from infected tissue. C) Survival curve shows that ML‐AMP2 significantly increased survival rates compared to fluconazole and saline controls. D) Body weight recovery also improved with ML‐AMP2 in a dose‐dependent manner. E) H&E‐stained sections of liver, lung, kidney, and spleen reveal reduced tissue damage and fungal burden in ML‐AMP2‐treated mice compared to saline. F) Time‐kill kinetics of ML‐AMP2 against *C. albicans* at different MIC multiples. G) Scanning electron microscopy (SEM) images showing intact fungal cells in PBS group, and severe membrane disruption in ML‐AMP2‐treated *C. albicans* (yellow = rupture, green = leakage, red = lysis). Reproduced with permission.^[^ ^341^ ^]^ Copyright 2025, Elsevier.

While Yin et al.^[^ ^340^ ^]^ employed deep learning to predict multiple bioactivity parameters and generate peptides de novo, a complementary approach was taken by Zhang et al.^[^ ^341^ ^]^ using a machine learning–driven selection pipeline to identify AMPs from a large sequence library. The study by Zhang et al.^[^ ^341^ ^]^ represents a major advancement in antifungal drug discovery by integrating ML for the de novo design of ML‐AMPs with broad‐spectrum antifungal activity. Among these, ML‐AMP2 stood out for its potent efficacy against both fluconazole‐sensitive and ‐resistant *C. albicans*. The peptides showed rapid fungicidal activity, strong inhibition of hyphae and biofilm formation, and low resistance‐inducing potential. Notably, ML‐AMP2 outperformed fluconazole in biofilm eradication assays and demonstrated comparable efficacy in an in vivo systemic candidiasis mouse model, achieving a 90% survival rate after 14 days of treatment (**Figure** **9**). This highlights the potential of ML‐generated AMPs not only to combat drug‐resistant fungal pathogens but also to function effectively in complex biological environments. A key contribution of this work lies in its demonstration of how machine learning accelerates the early discovery phase by enabling the rational prediction of AMP sequences with desired physicochemical and structural features. Rather than relying on exhaustive experimental screening, the researchers trained ML algorithms on curated peptide datasets to generate new AMP candidates, which were then synthesized and validated. This approach drastically reduces time, cost, and labor associated with traditional drug development pipelines. Furthermore, by integrating bioinformatics tools such as AlphaFold3, HeliQuest, and ExPASy, the authors ensured that only the most promising sequences were selected for synthesis and preclinical evaluation, thus improving translational efficiency.

The ML‐AMP platform demonstrated in this study offers a scalable and adaptable framework for antifungal peptide discovery. By streamlining candidate selection, predicting hemolytic toxicity, and modeling peptide–membrane interactions computationally, this approach minimizes empirical trial‐and‐error. Importantly, ML‐generated peptides like ML‐AMP2 were effective against biofilms—a major barrier in fungal treatment—and exhibited no significant toxicity in murine models.

### 6.3 Development of AMP‐Inspired Small Molecules with Antifungal Activity

Although AMPs have shown considerable promise as antifungal agents due to their broad‐spectrum activity and membrane‐targeting mechanisms, their clinical application is limited by key pharmacological challenges. These include susceptibility to proteolytic degradation, poor bioavailability, and short half‐life. In response, the field has shifted toward the development of peptidomimetics—synthetic or semi‐synthetic molecules designed to mimic the structural and functional properties of AMPs, while addressing their limitations through chemical modifications. These AMP‐inspired small molecules retain features essential for antifungal activity—such as amphipathicity and cationic charge—but incorporate non‐natural backbones or substitutions to improve stability, selectivity, and pharmacokinetics. Because of their reduced molecular weight and modifiable chemistry, they are often easier to synthesize, more resistant to enzymatic degradation, and suitable for oral delivery or scalable manufacturing.^[^ ^342^ ^]^ For example, FD10, derived from *P. brevitarsis* larvae, demonstrates selective antifungal activity and serves as a prototype for bioinspired design.^[^ ^343^ ^]^ Additionally, antimicrobial poly‐α‐amino acids offer favorable properties such as low toxicity, prolonged activity, and high structural flexibility.^[^ ^344^ ^]^

Among the most promising peptidomimetic scaffolds are β‐peptides, which replace the α‐amino acid backbone with β‐amino acids. This modification enables the formation of stable secondary structures (e.g., 14‐helices) that are highly resistant to proteases.^[^ ^345^ ^]^ In antifungal applications, β‐peptides have shown potent activity against *C. albicans* and have been specifically engineered to disrupt biofilm formation via membrane interactions and electrostatic binding.^[^ ^346^ ^]^ Their modular nature also allows tuning of charge distribution and hydrophobicity, enhancing selectivity and reducing cytotoxicity. Another relevant class includes peptoides (N‐substituted glycines), which are peptidomimetics characterized by side chains attached to the nitrogen rather than the α‐carbon. This structural change eliminates backbone hydrogen bonding, conferring exceptional resistance to proteolytic cleavage and improved membrane permeability. Peptoides have been explored for antifungal purposes due to their amphipathic helical conformations, and ongoing studies aim to optimize their selectivity against fungal membranes while minimizing hemolytic activity.^[^ ^347^ ^]^

Stapled peptides, which incorporate hydrocarbon bridges (“staples”) to stabilize α‐helical conformations, offer another strategy for enhancing peptide drug properties. These molecules exhibit increased helicity, membrane permeability, and resistance to enzymatic degradation. In antifungal research, stapled peptides are being evaluated for their ability to target intracellular fungal proteins and overcome delivery barriers associated with conventional AMPs.^[^ ^243^ ^]^

Hybrid systems such as α/β‐peptides, which combine natural α‐amino acids with β‐residues, provide an intermediate platform that balances biological activity with structural stability. These hybrids maintain the functional motifs of native AMPs while improving protease resistance and allowing precise control over conformation, Finally, foldamers, a broad class of non‐natural oligomers designed to adopt defined 3D shapes, have emerged as powerful tools for mimicking AMP‐like function. Their synthetic backbones can be programmed to form helices, sheets, or loops that replicate the topology and function of AMPs. Foldamers have shown promise in mimicking the membrane‐disruptive action of cationic AMPs, and are under investigation for their antifungal selectivity and structural tunability.^[^ ^345^, ^348^ ^]^ Altogether, the development of AMP‐inspired small molecules and peptidomimetics represents a promising and expanding frontier in antifungal drug discovery.

**Figure 10:**
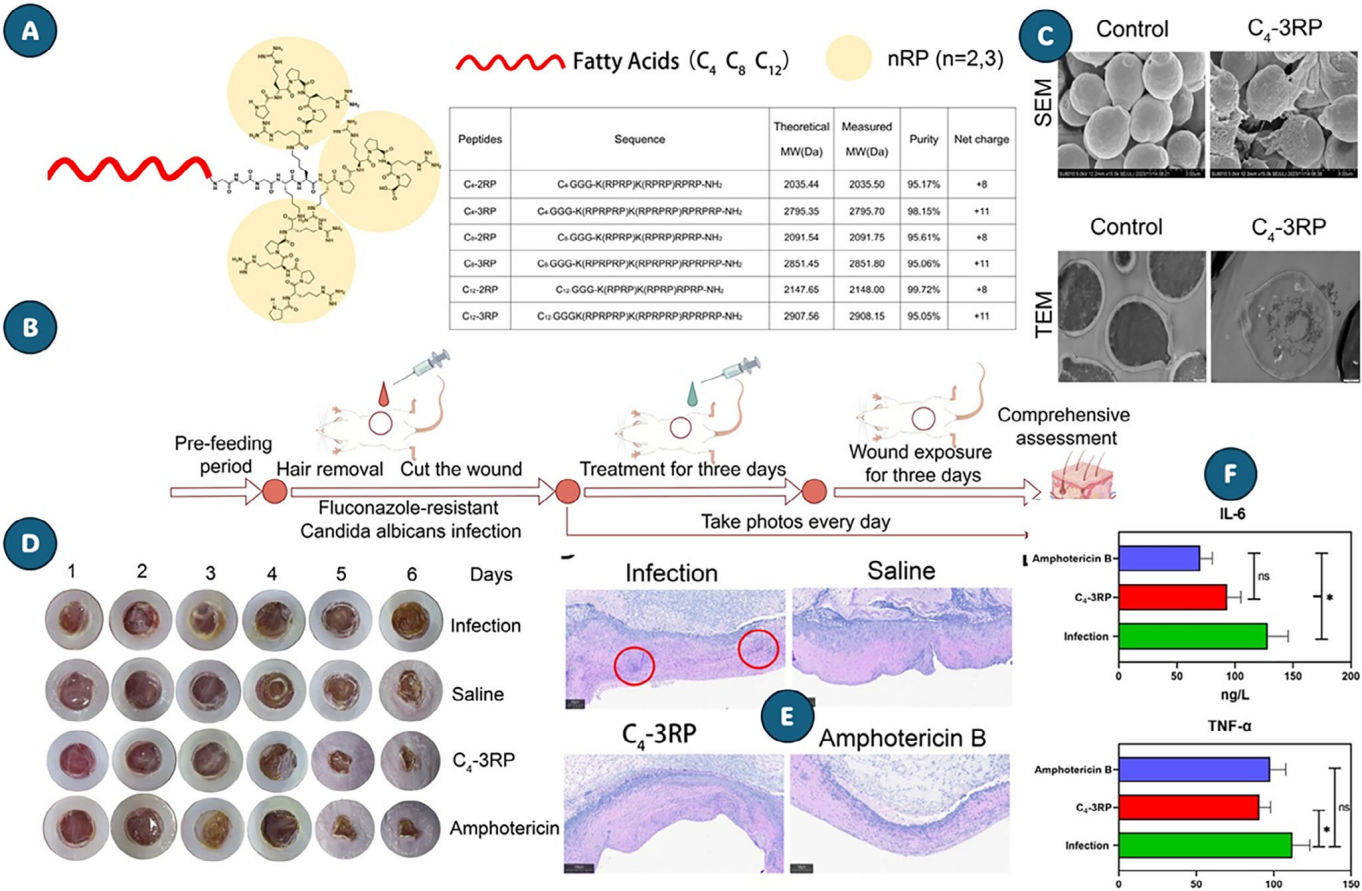
Structural design and in vivo antifungal efficacy of the dendritic peptidomimetic C_4_‐3RP against fluconazole‐resistant *C. albicans*. A) Schematic structure of C_4_‐3RP, consisting of a short‐chain fatty acid (C_4_) and three arginine–proline repeat branches. B) Peptide sequences and physicochemical properties of C_2_–C₁_2_ dendritic analogs. C) SEM and TEM images showing membrane disruption and cytoplasmic leakage in *C. albicans* treated with C_4_‐3RP. D) Wound healing progression in a murine model infected with fluconazole‐resistant *C. albicans* and treated topically with saline, C_4_‐3RP, or amphotericin B. E) Histological analysis reveals reduced tissue damage and improved re‐epithelialization in the C_4_‐3RP‐treated group. F) Cytokine levels of IL‐6 and TNF‐α in wound tissue, indicating immunomodulatory effects of C_4_‐3RP comparable to amphotericin B and significantly lower than untreated infection. Reproduced with permission.^[^ ^349^ ^]^ Copyright 2025, American Chemical Society.

The study by Li et al.^[^ ^349^ ^]^ presents a compelling advancement in antifungal drug development through the design of C4‐3RP, a dendritic peptidomimetic optimized to combat fluconazole‐resistant *C. albicans*. Unlike conventional AMPs, C4‐3RP combines a short‐chain fatty acid (C4) tail to enhance membrane interaction, with three Arg‐Pro repeats conferring high cationicity and protease resistance. This molecular architecture balances hydrophobicity and positive charge, enabling potent antifungal activity while maintaining low cytotoxicity. Notably, its random coil structure—rather than traditional α‐helix—remains stable in diverse environments, highlighting the importance of structural rigidity in therapeutic peptide design. The most impactful result of the study lies in the in vivo validation of C4‐3RP in a mouse model of drug‐resistant *C. albicans* skin infection (**Figure** **10**). Topical application of the peptide led to significant reduction in fungal burden (≈1.7 log_10_), comparable to amphotericin B, and markedly improved wound healing. C4‐3RP also attenuated the inflammatory response by downregulating IL‐6 and TNF‐α expression and reduced ROS accumulation, thereby preventing tissue damage while enhancing immune recovery. These outcomes underscore the therapeutic potential of C4‐3RP not only as a direct antifungal agent but also as an immunomodulatory molecule with reparative benefits. From a drug discovery standpoint, this study exemplifies the power of peptidomimetic strategies to overcome traditional AMP limitations such as enzymatic degradation, short half‐life, and limited biofilm efficacy. C4‐3RP's ability to inhibit and penetrate mature biofilms, kill internalized fungi, and resist degradation by proteases positions it as a robust candidate for clinical translation. The success of this dendritic design supports the continued exploration of modular, multivalent peptide mimics as next‐generation antifungals, especially against multidrug‐resistant pathogens where conventional agents fail.

### 6.4 Leveraging Natural Host Peptides for Antifungal Therapies and Immune Enhancement

Host defense peptides (HDPs) constitute a well‐defined subclass of AMPs that are endogenously produced by virtually all forms of life as part of the innate immune system. These peptides, typically short (10–50 amino acids), cationic, and amphipathic, are evolutionarily conserved molecules that serve as both direct antimicrobial agents and regulators of immune homeostasis.^[^ ^350^ ^]^ Unlike many synthetic AMPs designed purely for antimicrobial action, HDPs naturally function at the interface between microbial recognition and immune activation, making them particularly relevant in host–pathogen interactions.

HDPs include key families such as α‐defensins, β‐defensins, LL‐37, plectasin, indolicidin, protegrin‐1, cathelicidins, and histatins. These peptides display broad‐spectrum antimicrobial activity, including potent antifungal effects against *Candida* spp., *Aspergillus* spp., and *Cryptococcus* spp., primarily through membrane destabilization, pore formation, and interference with intracellular targets. However, their most distinctive attribute lies in their immunomodulatory capacity: HDPs can recruit and activate neutrophils, macrophages, and dendritic cells; modulate cytokine release (e.g., IL‐8, TNF‐α); enhance chemotaxis; and even promote wound healing and tissue regeneration.^[^ ^351^, ^352^ ^]^

In the context of fungal infections, HDPs play a dual role by directly inhibiting fungal growth and modulating host immune responses. For example, LL‐37 has been shown to suppress *C. albicans* biofilm formation while simultaneously enhancing monocyte and neutrophil responses.^[^ ^353^ ^]^ Similarly, histatin‐5, found in human saliva, exerts fungicidal activity via mitochondrial dysfunction and has been developed into derivatives (e.g., PAC113) currently under clinical evaluation for oral candidiasis.^[^ ^354^ ^]^

Beyond endogenous host peptides, recent research has highlighted the human microbiota as a valuable and underexplored source of AFPs with HDP‐like activity. Several commensal species, particularly within the *Firmicutes phylum*, produce short bioactive peptides such as mutanocyclin, reutericyclin, and 1‐acetyl‐β‐carboline, which have demonstrated potent inhibitory effects against *C. albicans* and related pathogens.^[^ ^355^ ^]^ These microbiota‐derived peptides often function through membrane disruption, modulation of filamentation, and interference with fungal signaling pathways, including the PKA–Sfl1 axis. Importantly, they also modulate the host immune response by shaping mucosal defense and enhancing epithelial resilience.

Despite their therapeutic potential, clinical translation of HDPs remains challenging due to several inherent limitations: rapid proteolytic degradation, possible cytotoxicity at high doses, low bioavailability, and high manufacturing costs. To address these issues, advanced delivery and stabilization strategies are under investigation. These include PEGylation, lipidation, cyclization, and conjugation with synthetic polymers or nanoparticles to enhance half‐life and reduce immunogenicity. Furthermore, bioinformatics and rational design approaches are now widely used to optimize HDP sequences for selective toxicity and improved therapeutic index.

Recent research has also focused on developing next‐generation HDPs, incorporating unnatural amino acids, backbone modifications, and non‐peptidic mimetics that preserve immunomodulatory function while enhancing drug‐like properties. Notably, HDAC inhibitors and epigenetic modulators have been shown to upregulate endogenous HDP expression, offering an indirect therapeutic strategy by harnessing the body's own antimicrobial arsenal.^[^ ^356^ ^]^

Several preclinical models have shown that HDPs, such as defensins and cathelicidins, enhance the efficacy of azoles and echinocandins by disrupting fungal membranes and impairing stress adaptation mechanisms.^[^ ^357^, ^358^, ^359^ ^]^ This synergy opens the door to dose‐sparing strategies, reducing toxicity and improving outcomes in invasive mycoses. Altogether, HDPs represent a promising yet underutilized class of antifungal agents that uniquely combine direct antimicrobial activity with immunological enhancement. Their integration into clinical practice, either as standalone therapeutics, adjuncts, or immune‐priming agents, could significantly advance the management of systemic fungal infections, especially amid rising antifungal resistance and immunosuppressive therapies.

## 7 Clinical Translation and Current Trials of AMP‐Based Antifungals

Traditional antifungal medications include azoles (e.g., isavuconazole, voriconazole, fluconazole), polyenes (like amphotericin B), echinocandins (such as caspofungin and micafungin), allylamines (e.g., terbinafine), and antimetabolites (e.g., flucytosine).^[^ ^360^ ^]^ Despite their widespread use, issues related to limited bioavailability, host toxicity, and emerging resistance have driven the pursuit of next‐generation antifungal agents and optimized formulations. One such advancement is SUBA‐itraconazole, a reformulated version of itraconazole that improves absorption and achieves bioavailability exceeding 100% under physiological conditions.^[^ ^361^ ^]^ Other investigational agents include rezafungin, shown to be active against *C. auris*,^[^ ^362^ ^]^ and compounds such as ibrexafungerp and olorofim, both of which are undergoing phase II–III clinical trials. Among the most promising candidates, fosmanogepix is currently being evaluated for infections caused by *Candida, Aspergillus, Fusarium, Rhizopus*, and *Coccidioides* species.^[^ ^363^ ^]^ Ibrexafungerp has completed trials targeting *Candida* spp., *Aspergillus* spp., and *Pneumocystis jirovecii*. New formulations of amphotericin B and novel compounds like olorofim are also being explored for activity against difficult‐to‐treat fungal pathogens, including *Histoplasma capsulatum*. In parallel, AMP‐based therapies have begun to advance into both preclinical and early clinical stages, reinforcing interest in these molecules not only as stand‐alone therapeutics but also as adjuncts to existing treatments. These peptides exhibit distinct mechanisms of action, and their development faces several regulatory challenges. Prior to approval, AMP candidates must complete a rigorous pipeline of in vitro and in vivo testing, followed by safety and efficacy evaluations in human clinical trials.^[^ ^364^ ^]^

Regulatory bodies such as the FDA, European Medicines Agency, and Japan's Pharmaceuticals and Medical Devices Agency oversee these processes, ensuring compliance with standards related to manufacturing, consistency, and safety.^[^ ^234^, ^265^ ^]^ Agencies such as the National Institute of Allergy and Infectious Diseases provide crucial support for antifungal AMP development through funding and validation efforts.^[^ ^365^ ^]^ AMPs must also overcome additional obstacles, including peptide degradation, cytotoxicity, and challenges related to scaling up production. Establishing reproducible manufacturing protocols and ensuring the stability of AMP formulations are essential steps. Identification of fungal‐specific antigens, such as enolase and phosphoglycerate kinase in *C. auris*, may enable the design of dual‐function AMPs with diagnostic and therapeutic applications. Several AMP candidates have progressed into clinical evaluation with encouraging outcomes. For example, Novexatin (NP213), a cyclic peptide derived from HDPs, showed promising results in phase IIb trials for onychomycosis, combining low systemic toxicity with favorable patient tolerability. Likewise, PAC113, a derivative of salivary histatin 5, demonstrated therapeutic benefit in HIV‐positive individuals with oral candidiasis. The peptide hLF1‐11, derived from human lactoferrin, has shown safety and preliminary efficacy in early‐phase clinical trials involving patients undergoing stem cell transplantation. Its use in topical or mucosal applications appears promising due to reduced enzymatic degradation (**Table** **3**). Nonetheless, clinical translation of antifungal AMPs remains limited, highlighting the need for targeted strategies to overcome pharmacokinetic barriers, ensure regulatory alignment, and enable scalable therapeutic development.

**Table 3:** Selected AMPs with antifungal activity currently in preclinical or clinical development. Source: http://dramp.cpu‐bioinfor.org/ (accessed June 2025).

AMP	Description	Medical application	Phase	Antifungal activity (µM)		DRAMP ID/link	References
hLF1‐11	Human lactoferrin 1–11	Fungal and bacterial infections	phase I‐II	*C. albicans*	4 – 8	DRAMP18068	[366]
Mycoprex	Extracted from insects	Fungal infections	phase III	*C. albicans* *C. tropicalis* *C. parapsilosis* *C. krusei* *Aspergillus fumigatus* *A. flavus* *A. terreus* *Cryptococcus neoformans* *Fusarium solani*	0.32–0.64 0.64 0.16–0.32 0.32 1.28–2.56 1.28 0.64–1.28 1.56 0.64	DRAMP18071	[367]
Heliomocin variants (ETD151)	44‐amino acid antifungal peptide	fungal infections	Preclinical	*C. albicans* *C. Tropicalis* *C. parapsilosis* *C. Krusei* *Aspergillus Fumigatus* *A Flavus* *A Terreus* *Cryptococcus neoformans* *Fusarium solani*	0.32 – 0.64 0.64 0.16 – 0.32 0.32 1.28 – 2.56 1.28 0.64 – 1.28 1.56 0.64	DRAMP18072	[367]
PAC113	Extracted protein found in human saliva	Oral candidiasis	phase IIb	‐	‐	DRAMP18081 http://dramp.cpu‐bioinfor.org/browse/clinical‐information.php?id = DRAMP18081	NR
CZEN‐002	derived from alpha‐Melanocyte‐Stimulating Hormone	fungal infections (*Candida albicans*)	phase IIb	‐	‐	DRAMP18083	[368]
NVXT (NovexatinNP213)	Derived from arginine platform peptide	Fungal nail infection Onychomycosis	phase IIb	*T. rubrum*	2000 mg/l	DRAMP18157	[369]
HXP124	Plant defensin	Fungal nail infection (onychomycosis)	phase II	‐	‐	DRAMP29319	NR
Gramicidin S	biosynthesized from gramicidin in Bacillus brevis	Potent against gram‐negative and gram‐positive bacteria and fungi	In Market	‐	‐	DRAMP29332	NR
Tyrothricin	obtained from Bacillus brevis.	Antibacterial, Antifungal	In Market	‐	‐	DRAMP29329	NR
HB1275	A lipohexapeptide	Antifungal Activity	Preclinical	‐	‐	DRAMP18078	NR
Plectasin	fungal defensin	Antibacterial	Phase I	.	.	DRAMP18080	NR
Histatin	Found the saliva of man and some higher primates	Antifungal	Phase II‐III	*C. albicans*	0.6 a 0.8	DRAMP18061 http://dramp.cpu‐bioinfor.org/browse/clinical‐information.php?id = DRAMP18061	[370]

NR: No references reported on the website.

## 8 Conclusion and Final Remarks

AMPs, including AFPs, offer a compelling alternative to conventional therapies in the face of rising resistance among WHO‐priority fungal pathogens. Their ability to act through multiple mechanisms—membrane disruption, immune modulation, and intracellular targeting—makes them uniquely suited to address the complexity of fungal infections. However, the field remains limited by fragmented development pipelines, reliance on in vitro screening, and insufficient integration of fungal‐specific biology into AMP design. Many candidates fail in vivo due to poor selectivity, short half‐life, or toxicity, highlighting the need for more predictive models and clinically relevant delivery systems. To address these gaps, we propose a shift toward target‐guided peptide engineering, integrating insights from fungal lipidomics and immunoproteomics. Designing AMPs that selectively interact with fungal‐specific lipids—such as glucosylceramide or phosphatidic acid—and evasion mechanisms like β‐glucan masking may improve precision while limiting host damage. In parallel, modular peptide architectures can allow for tailored activity, with distinct regions optimized for membrane binding, immune activation, or intracellular delivery. Future studies should move beyond isolated efficacy to explore AMP combinations, protease‐sensitive carriers, and in vivo models that reflect clinical complexity, including immune suppression and biofilm formation. Repositioning AMPs will not be achieved through incremental advances alone—it will require new frameworks that align molecular design with translational goals. With such a shift, AMPs could redefine how we approach fungal therapeutics in the coming decade.

## 9 Significance Statement

The global burden of invasive fungal infections continues to rise, exacerbated by limited antifungal drug options, high resistance rates, and the emergence of multidrug‐resistant species listed by the World Health Organization (e.g., *Candida auris, Aspergillus fumigatus*). This review underscores the promising role of antimicrobial peptides (AMPs) as next‐generation therapeutics, offering rapid, multi‐target antifungal activity with reduced resistance development. By integrating structural design strategies, nanotechnology, and computational tools, the paper highlights innovative approaches to overcome current therapeutic limitations and inform future clinical development of AMP‐based antifungal treatments.

## Conflict of Interest

The authors declare no conflict of interest.
